# Combined analysis of single-cell sequencing and bulk transcriptome sequencing reveals new mechanisms for non-healing diabetic foot ulcers

**DOI:** 10.1371/journal.pone.0306248

**Published:** 2024-07-01

**Authors:** Ran Chen, Lijun Zou

**Affiliations:** Department of Wound Repair Surgery, Liyuan Hospital, Tongji Medical College, Huazhong University of Science and Technology, Wuhan, Hubei, China; PLOS: Public Library of Science, UNITED STATES

## Abstract

Diabetic foot ulcers (DFUs) pose a significant challenge in diabetes care. Yet, a comprehensive understanding of the underlying biological disparities between healing and non-healing DFUs remains elusive. We conducted bioinformatics analysis of publicly available transcriptome sequencing data in an attempt to elucidate these differences. Our analysis encompassed differential analysis to unveil shifts in cell composition and gene expression profiles between non-healing and healing DFUs. Cell communication alterations were explored employing the Cellchat R package. Pseudotime analysis and cytoTRACE allowed us to dissect the heterogeneity within fibroblast subpopulations. Our findings unveiled disruptions in various cell types, localized low-grade inflammation, compromised systemic antigen processing and presentation, and extensive extracellular matrix signaling disarray in non-healing DFU patients. Some of these anomalies partially reverted in healing DFUs, particularly within the abnormal ECM-receptor signaling pathway. Furthermore, we distinguished distinct fibroblast subpopulations in non-healing and healing DFUs, each with unique biological functions. Healing-associated fibroblasts exhibited heightened extracellular matrix (ECM) remodeling and a robust wound healing response, while non-healing-associated fibroblasts showed signs of cellular senescence and complement activation, among other characteristics. This analysis offers profound insights into the wound healing microenvironment, identifies pivotal cell types for DFU healing promotion, and reveals potential therapeutic targets for DFU management.

## Introduction

The incidence of diabetes mellitus (DM) is increasing every year, and various complications pose a threat to patients’ lives. Diabetic foot ulcers (DFUs) are one of the most common and severe complications of diabetes, as the healing of foot wounds is impaired in diabetic patients. DFUs are associated with decreased quality of life, lower limb amputations, hospitalizations, as well as high morbidity and mortality rates [1]. Only 30% of chronic diabetic foot ulcers can heal after 20 weeks of standard treatment [2].

Extensive research has reported the involvement of various cell types, including endothelial cells, [3] fibroblasts [4], keratinocytes [5], and immune cells [6], in the healing process of DFUs. However, the cellular composition differences between healing and non-healing DFUs in the context of diabetes are still unclear. Further research is needed to explore the molecular-cellular-biological differences between healing and non-healing DFUs. Analyzing the cellular and biological differences between DFU healing and non-healing patients can greatly enhance our understanding of the pathogenesis/healing mechanisms of DFUs.

Bulk sequencing (Bulk-Seq) can observe the average expression levels of tissue or cell RNA but overlooks the heterogeneity between cells or tissues. Single-cell RNA sequencing (scRNA-seq) overcomes this limitation. scRNA-seq analysis, by profiling the transcriptomes of individual cells from different tissues, provides a deep understanding of cellular function and the pathophysiology of diseases. In this study, high-quality DFUs-related scRNA-seq data sets published by Theocharidis et al., [7] as well as DFUs-related bulk transcriptome sequencing data sets published by Andrew P Sawaya et al. [8], were selected to investigate the biological differences between healing and non-healing DFUs.

Specifically, we explored the biological differences between healing and non-healing DFUs in terms of cell type composition, cell and tissue-specific molecular programs, cell-cell communication networks, and heterogeneity of fibroblast subpopulations. We observed widespread disruptions in all these aspects in non-healing DFUs, with partial recovery of these aspects observed in healing DFUs. Overall, this study serves as an important complement to the research conducted by the aforementioned scholars.

## Methods and materials

### 1. Dataset selection and bulk dataset pre-processing

To investigate the transcriptional differences between diabetes patients with and without foot ulcers in foot (ulcer) skin or blood samples, we selected the bulk transcriptome dataset GSE134431 [8] and the single-cell transcriptome dataset GSE165816 [7] from Gene Expression Omnibus (GEO) repository. All data collection, processing, and sharing comply with the ethical requirements and privacy policies of the original data providers. Therefore, this study does not involve new ethical approval or participant consent. Due to ethical and moral considerations, the uploaders did not provide specific clinical information about the samples. After excluding irrelevant samples, GSE134431 included 8 foot skin samples from diabetes patients without foot ulcers, 7 foot skin (ulcer) samples from healing diabetic foot ulcers, and 6 foot skin (ulcer) samples from non-healing diabetic foot ulcers. GSE165816 included 8 foot skin samples from diabetes patients without foot ulcers, 2 peripheral blood samples from diabetes patients without foot ulcers, 9 foot skin (ulcer) samples from healing diabetic foot ulcers, 3 peripheral blood samples from healing diabetic foot ulcers, 5 foot skin (ulcer) samples from non-healing diabetic foot ulcers, and 2 peripheral blood samples from non-healing diabetic foot ulcers. To exclude genes with expression changes occurring during acute wound healing, we selected the acute wound healing-related gene chip dataset GSE28914 [9], which included 8 skin samples from day 0 of wound healing, 6 immediate post-biopsy skin samples (day 1), 6 skin samples from day 3 of wound healing, and 5 skin samples from day 7 of wound healing. Detailed information about the datasets can be found in S1 Table. The gene chip datasets or bulk sequencing datasets were normalized and subjected to PCA clustering analysis, as shown in S1A–S1D Fig.

### 2. Single-cell RNA sequencing data analysis

#### 2.1 preprocessing, filtering, and normalization

Single cell analyses were performed using “Seurat v4” [10]. Single-cell gene expression data of all patients were merged, and transcriptomes were filtered for cells with 500–10,000 genes detected, 1000–100,000 UMIs counted, fraction of mitochondrial reads <30%, and fraction of hemoglobin reads <5%. After filtering, UMI counts were variance-stabilized using scTransform with 3000 variable features [11], while regressing out number of UMIs and fraction of mitochondrial reads.

#### 2.2 Clustering and cell type annotation

Unsupervised principal component analysis (PCA) was conducted to identify the principal components capturing the variation in gene expression and capturing the maximum variance across the samples. These principal components were then used as input for Uniform Manifold Approximation and Projection (UMAP) analysis to determine the overall relationship among the cells. Cells with similar transcriptomic features were clustered together, and these clusters were further annotated to different cell types based on the expression of specific, well-established cell marker transcripts. The cell type markers were compiled from studies by Cellmarker [12], Llorenç Solé-Boldo, [13] Theocharidis, G [7], among others. The final set of identified cell markers can be found in S2 Table. Comparative analysis of the single-cell landscape of diabetic skin without ulcers and diabetic wounds was performed using split UMAP plots to determine the heterogeneity of cell populations and the abundance of different cell types.

### 3. Analysis of cellular composition variation

The cell counts of each cell type in different groups (DS, DFUNH, DFUH) were determined, and the cell counts were divided by the total number of cells in the same group to calculate the cell type proportions. Based on these proportions, the percentages of specific cell types were calculated for each group. Then, the Log2FC (log fold change) between the DFUNH group and the DS group were calculated to identify cell types that changed in non-healing wounds of diabetes (|Log2FC| > 0.1). Similarly, the Log2FC between the DFUH group and the DFUNH group were calculated to identify cell types that changed in healing wounds (|Log2FC| > 0.1). Therefore, "rescue cell types" were defined as cell types that changed in non-healing wounds and were rescued in healing wounds. Additionally, the proportions of each cell type in each sample were calculated, and based on these proportions, the changes in cell type abundances between clinical groups were evaluated. A t-test was performed to assess the significance of cell type abundance changes between clinical groups (p-value < 0.05).

### 4. Analysis of cell-specific differentially expressed gene networks

The "FindMarkers" function in the Seurat package (version 4.3.0) was utilized to perform differential expression analysis of each cell type between different groups (DFUNH/DS and DFUH/DFUNH) using the Wilcoxon rank-sum test. Prior to conducting the differential expression analysis, cell types that were missing or had fewer than three cells in the comparison groups (DFUNH/DS and DFUH/DFUNH) were filtered out. Consequently, platelets, plasma-like dendritic cells, HSPCs (hematopoietic stem and progenitor cells), granulocytes, and granulocyte-monocyte progenitors were excluded from the peripheral blood. First, differentially expressed genes (DEGs) between the DFUNH and DS groups were identified, generating a dataset of DEGs associated with non-healing (unhealing DEGs) (|LogFC| > 0.25, adjusted p-value < 0.01). Subsequently, DEGs between the DFUH and DFUNH groups were identified, generating a dataset of DEGs associated with healing (healing DEGs) (|LogFC| > 0.25, adjusted p-value < 0.01). Based on the aforementioned results, "rescue DEGs" were defined as genes that were downregulated during the non-healing process and upregulated during the healing process (rescue upregulated DEGs), or genes that were upregulated during the non-healing process and downregulated during the healing process (rescue downregulated DEGs). Next, a cell-DEG network and a rose plot were constructed based on the aforementioned DEGs (unhealing DEGs, healing DEGs, and rescue DEGs) using single-cell RNA sequencing data, integrating the tissue and cell type sources of DEGs. The cell-DEG network was visualized using Cytoscape software (version 3.8.2) [14].

### 5. Short Time-series Expression Miner (STEM) analyses

Short Time-series Expression Miner (STEM) is a Java program used for clustering, comparing, and visualizing short time-series gene expression data from microarray experiments with 8 or fewer time points. In this study, the dynamic gene expression clusters associated with acute non-diabetic wound healing and diabetic wound healing were identified using the Short Time-series Expression Miner software (version 1.3.8) [15]. The dataset GSE28914 comprised four groups of samples at different time points during acute wound healing. For each group, the gene expression values were averaged to construct a matrix of mean expressions. Similarly, in the dataset GSE134431, three groups of skin wound samples in different states were used to construct a mean expression matrix using the same approach. The STEM clustering method was selected and other options were set as default. The gene expression profiles were clustered based on statistically significant values (P-value < 0.05). Genes that exhibit an increasing trend followed by a decreasing trend during the wound healing process are referred to as "rescue down" genes. On the other hand, genes that show a decreasing trend followed by an increasing trend are referred to as "rescue up" genes.

### 6. Gene Ontology (GO) and Protein-Protein Interaction (PPI) analysis

Gene Ontology (GO) and Protein-Protein Interaction (PPI) analyses were performed using the Metascape website. Default parameters were used for the analysis. For GO enrichment analysis, the minimum overlap was set to 3, the p-value cutoff was set to 0.01, and the minimum enrichment was set to 1.5. For PPI analysis, the minimum network size was set to 3, the maximum network size was set to 500, and physical core was used. The differential gene enrichment analysis among fibroblast subgroups was performed using the DAVID online website, and the enrichment results were visualized using R language.

### 7. Gene Set Enrichment Analysis (GSEA) analysis

Gene Set Enrichment Analysis (GSEA) is a common framework that integrates information from gene expression profiles into pathway or signature summaries. We used the GSEA method to estimate the activity of gene sets from bulk and single-cell RNA sequencing data. Specifically, for single-cell data, the "findmarkers" function was used to calculate differentially expressed genes between groups, with the filtering criteria set as adjusted p-value less than 0.05 and an average log2 fold change (avg_log2FC) greater than 0.5. The GSEA R package was applied to analyze the enrichment scores of the interested gene sets. Gene sets were considered significant when the adjusted p-value was less than 0.05 and the normalized enrichment score (NES) was greater than or equal to 1. For bulk data, the "limma" package in the online tool NetworkAnalyst was used to analyze the differentially expressed genes between the two groups (DFUNH vs DS, DFUH vs DFUNH). The GSEA R package was then used to analyze the enrichment scores of the interested gene sets, with the same filtering criteria as for the single-cell data. The gene sets were selected from the GSEA-msigDB database, specifically from c2.cp.v7.4.symbols.gmt (KEGG) and c5.go.bp.v7.4.symbols.gmt (GO-BP).

### 8. Cell-cell communication analysis

The cell-cell communication analysis was conducted using the CellChat [16] software package (version 1.1.0) in R language. Skin cells were annotated based on their cell types, including differentiated keratinocyte (DiffKera), basal keratinocyte (BasalKera), smooth muscle cells (SMCs), fibroblast (Fibro), vascular endothelial cells (VasEndo), T lymphocyte (T_lympho), natural killer cell (NK), M1 macrophage (M1), M2 macrophage (M2), melanocytes/schwann cell (Melano_Schwann), lymphatic endothelial cells (LymphEndo), B lymphocyte (B_lympho), plasma cell (Plasma_cell), mast cell (Mast_cell), and merkel cell (Merkel_cell). The "createCellChat" function was used to create a CellChat object. After annotating the objects with relevant labels and identifying the differentially expressed genes, the "computeCommunProb" function was used to infer the communication probabilities between cells. The "computeCommunProbPathway" function was used to generate intercellular communication for each cell signaling pathway. The "netVisual_chord_gene" function was used to generate the visualization. The "subsetCommunication" function was employed to determine the upregulated or downregulated ligand-receptor (LR) pairs in the cell communication network. The filtering criteria were as follows: upregulated LR: ligand.logFC > = 0.2, receptor.logFC > = 0.2; downregulated LR: ligand.logFC < = -0.2, receptor.logFC < = -0.2.

### 9. Single-cell trajectory analysis and CytoTRACE analysis

In this study, we employed the Monocle 3 [17] algorithm to perform single-cell trajectory analysis and detected gene expression changes in fibroblast subtypes during cellular transitions. Monocle 3 utilizes a single-cell trajectory analysis strategy that leverages algorithms to learn gene expression changes occurring in each cell during dynamic biological processes. By constructing a global "trajectory" of gene expression changes, each cell can be placed in the appropriate position within a reduced-dimensional space for trajectory analysis. Additionally, CytoTRACE [18] analysis was performed to predict the differentiation status of cells in the single-cell RNA sequencing data, enabling the identification of cell subtypes located at the beginning of the cell trajectory.

### 10. Selection of differentially expressed genes in fibroblast subpopulations

The FindMarker function was used to identify differentially expressed genes between the "good" and "bad" cells. The selection criteria were an absolute log fold change (logFC) greater than or equal to 0.25, an adjusted p-value less than 0.01, and the Wilcoxon test was employed.

### 11. Curve analysis of Receiver Operating Characteristics (ROC)

We used the pROC function in the R package to create Receiver Operating Characteristic (ROC) curves to determine the area under the curve (AUC) for screening signature genes and evaluating their diagnostic value. The potential of its role as a molecular biomarker was evaluated based on the value of the AUC [19].

## Results

### 1. Construction of a single-cell atlas of diabetic foot ulcers

**Fig 1:**
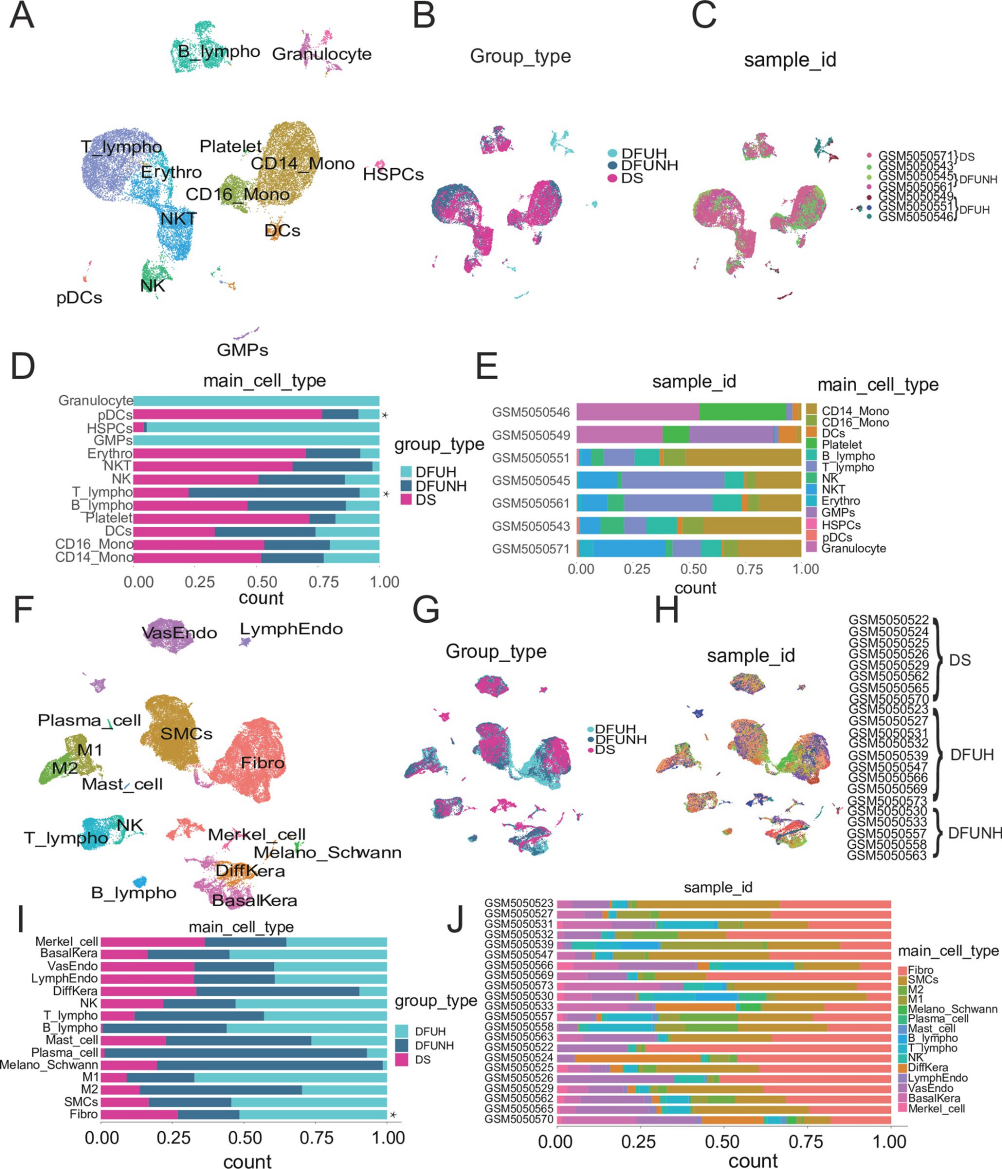
Single-cell RNA sequencing revealed identification and characterization of cell types in diabetic foot ulcers (DFUs) skin and peripheral blood. (A, F) Uniform Manifold Approximation and Projection (UMAP) embeddings generated from datasets containing 52335 cells (A) and 23567 cells (F), respectively. Cells are colored based on orthogonal-generated clusters and annotated manually according to their cell types (Skin cell types: smooth muscle cells, SMCs; fibroblasts, Fibro; vascular endothelial cells, VasEndo; T lymphocytes, T_lympho; differentiated keratinocytes, Diffkera; basal keratinocytes, BasalKera; natural killer cells, NK; M1 macrophages, M1; M2 macrophages, M2; melanocytes and Schwann cells, Melano_Schwann; lymphatic endothelial cells, LymphEndo; B lymphocytes, B_lympho; plasma cells, Plasma_cell; mast cells, Mast_cell; and Merkel cells, merkel_cell. Typical cell types in peripheral blood: natural killer cells, NK; natural killer T cells, NKT; Erythrocytes, Erythro; dendritic cells, DC; T lymphocytes, T_lympho; CD14^+^ monocytes, CD14_Mono; CD16^+^ monocytes, CD16_Mono; B lymphocytes, B_lympho; plasmacytoid dendritic cells, pDCs; platelets; granulocytes; granulocyte-monocyte progenitors, GMPs; and hematopoietic stem and progenitor cells, HSPCs). (B, G) UMAP embeddings of peripheral blood cells (B) and skin cells (G), colored based on clinical group, from all analyzed samples. (C, H) UMAP embeddings of peripheral blood cells (C) and skin cells (H), colored based on sample source, from all analyzed samples. (D, I) The stacked bar charts display the proportions of different cell types in the three clinical groups of peripheral blood (D) and skin (I). Light blue: DFU-Healers, dark blue: DFU-non-Healers, purple-red: patients with diabetes without foot ulcers. Cell types with significant differences between clinical groups are marked with an asterisk. The differences in proportions of each cell type in each group are shown in S4 Fig. (E, J) The stacked bar charts display the proportions of different cell types in each analyzed sample of peripheral blood (E) and skin (J). Different colors represent different cell types.

In order to identify local and systemic factors associated with DFUs healing, we performed scRNA-Seq analysis on foot skin samples (n = 8) and peripheral blood samples (n = 2) from patients with diabetes without foot ulcers, foot ulcer samples (n = 9) and peripheral blood samples (n = 3) from patients with healed DFUs, and foot ulcer samples (n = 5) and peripheral blood samples (n = 2) from patients with non-healed DFUs. After quality control (S2A and S2B Fig), we analyzed 52335 skin cells and 23567 peripheral blood cells, respectively, and created a gene expression matrix for each cell. We used these matrixs to perform dimensionality reduction via UMAP and graph-based clustering, identifying 29 and 17 orthogonal cell clusters in the skin and peripheral blood, respectively (S3A, S3B, S3E and S3F Fig). The expression of established cell-specific marker genes aided in annotating these cell clusters into 15 and 13 distinct cell types, respectively (Fig 1A and 1F and S3C, S3D, S3G and S3H Fig). Typical cell types in the skin included smooth muscle cells (SMCs) (TAGLN^+^, ACTA2^+^), fibroblasts (Fibro) (DCN^+^, CFD^+^), vascular endothelial cells (VasEndo) (ACKR1^+^), T lymphocytes (T_lympho) (CD3D^+^), differentiated keratinocytes (Diffkera) (KRT1^+^, KRT10^+^), basal keratinocytes (BasalKera) (KRT5^+^, KRT14^+^), natural killer cells (NK) (CCL5^+^, GZMB^+^), M1 macrophages (M1) (IL1B^+^), M2 macrophages (M2) (CD163^+^), melanocytes and Schwann cells (Melano_Schwann) (MLANA^+^, CDH19^+^), lymphatic endothelial cells (LymphEndo) (CCL21^+^), B lymphocytes (B_lympho) (CD79A^+^, MS4A1^+^), plasma cells (plasma_cell) (MZB1^+^), mast cells (Mast_cell) (TPSAB1^+^) and Merkel cells (Merkel_cell) (KRT18^+^) (Fig 1F and S3C, S3D Fig). Typical cell types in peripheral blood included natural killer cells (NK) (CCL5^+^, GZMB^+^), natural killer T cells (NKT) (CD3D^+^, CCL5^+^), Erythrocytes (Erythro) (HBB^+^), dendritic cells (DCs) (GZMB^+^, IRF8^+^), T lymphocytes (T_lympho) (CD3D^+^), CD14^+^ monocytes (CD14_Mono) (CD14^+^, S100A9^+^), CD16^+^ monocytes (CD16_Mono) (FCGR3A^+^, MS4A7^+^), B lymphocytes (B_lympho) (CD79A^+^, MS4A1^+^), plasmacytoid dendritic cells (pDCs) (LILRA4^+^), platelets (ITGA2B^+^, ITGB3^+^, SELP^+^, PPBP^+^), granulocytes (CD63^+^, ENPP3^+^, CEACAM8^+^), granulocyte-monocyte progenitors (ADK^+^, ALDH4A1^+^, ANXA1^+^), and hematopoietic stem and progenitor cells (HSPCs) (CD34^+^) (Fig 1A and S3G, S3H Fig). Comparison and statistical analysis of cell type abundance demonstrated significant differences in the concentrations between different clinical groups and each sample (Fig 1B–1E and 1G–1J). Statistical analysis of cell abundance revealed significant differences in Fibro in skin tissue and plasmacytoid dendritic cells and T cells in blood between clinical groups (S4A and S4B Fig).

### 2. Reconstruction of the cellular ecosystem for diabetic wound healing

**Fig 2:**
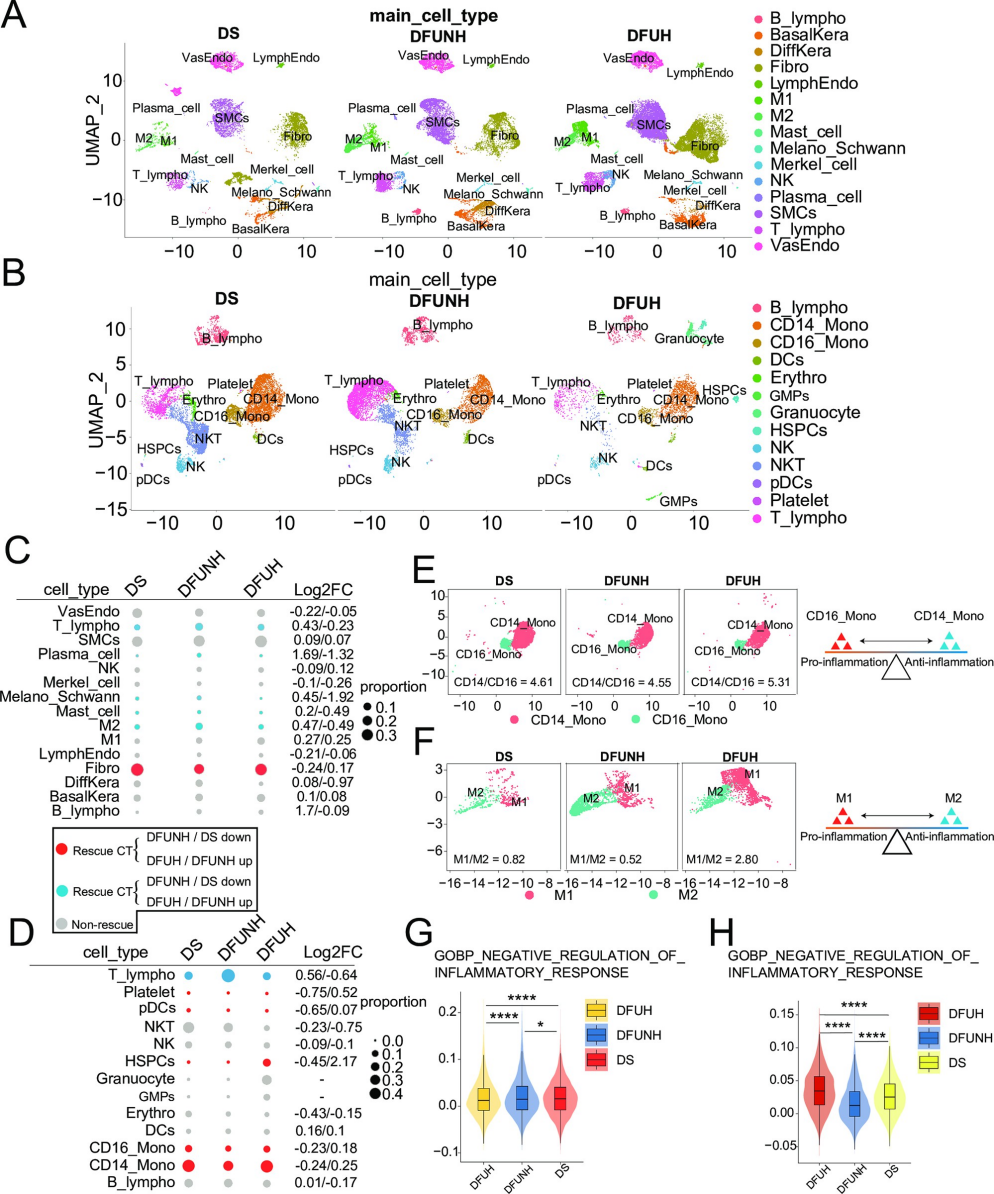
Dynamic composition changes of cell types during the diabetic wound healing process. (A-B) UMAP plots showing the composition changes of various cell types in the skin (A) and peripheral blood (B) of the DS, DFUNH, and DFUH groups. (C-D) Relative changes in cell proportions among the three groups in skin (C) and periheral blood (D). The numbers on the right indicate the logarithmic fold change in cell proportions (DFUNH/DS and DFUH/DFUNH). The cell types marked in red are downregulated in non-healing wounds and upregulated in healing wounds, while the cell types marked in blue are upregulated in non-healing wounds and downregulated in healing wounds. Gray indicates cell types with no significant proportional changes. (E) UMAP plot displaying the changes in CD14 /CD16 monocyte cell proportions. (F) UMAP plot showing the changes in M1/M2 macrophage proportions. (G-H) Differences in the scores of negative regulation of inflammatory response in local wound and peripheral blood among the three clinical groups. * indicates p<0.05. **** indicates p<0.0001.

The cells may exhibit continuous variations or minor differences between different clinical groups, which may be overlooked at a statistically significant level. To further delineate the dynamic and subtle changes in cell composition in diabetic skin, diabetic non-healing wounds (DFUNH), and diabetic healing wounds (DFUH), we compared the proportional changes of each cell type in skin wound tissues and peripheral blood between the DS, DFUNH, and DFUH groups, respectively. Fig 2 demonstrates the cell types that exhibit changes in proportion in non-healing wounds and undergo reversal in healing wounds (see Methods). Overall, we observed changes in the proportions of various cell types in the DFUNH group compared to the DS group, many of which were rescued in the DFUH group (Fig 2A–2D). For example, the proportions of T lymphocytes, plasma cells, melanocytes, mast cells, M2 cells in skin and T lymphocytes in peripheral blood increased in the DFUNH group, while they decreased in the DFUH group. Similarly, the proportions of fibroblasts in skin and CD14 monocytes, CD16 monocytes, hematopoietic stem and progenitor cells (HSPCs), plasmacytoid dendritic cells, and platelets in periheral blood decreased in the DFUNH group, while they increased in DFUH group (Fig 2A–2D). These changes in cell proportions may have negative or positive effects on wound healing. Fibroblasts are an important cell type in wound healing, involved in different stages of the healing process. They produce collagen and other matrix proteins during the healing process, providing structural support and the necessary matrix for wound repair. Fibroblasts are also involved in angiogenesis, promoting the formation of new blood vessels by secreting angiogenic factors, thus improving the blood supply to the wound site [20]. However, diabetes affects the function and activity of fibroblasts. Under high glucose conditions, the proliferation and migration abilities of fibroblasts decrease, and the quality of collagen production is reduced [4]. The specific role of plasmacytoid dendritic cells (pDCs) in the wound healing process in diabetes is not yet clear. pDCs play an important role in immune responses, primarily involved in the immune response to viral infections and regulating the immune system by producing interferon-alpha (IFN-alpha) in large quantities [21]. We speculate that pDCs may affect the healing of diabetic wounds through the aforementioned mechanisms. In a thymectomized rat model, depleted CD8 T cells have a positive impact on wound healing, while CD4 T cell depletion has a negative effect [22]. To explore the differences in CD4+ and CD8+ T cells between the DS, DFUNH, and DFUH groups, we further divided peripheral blood T cells into CD4+ and CD8+ subgroups and analyzed the changes in the composition of CD4+ and CD8+ cells among the three groups. Our study results showed a downregulation of CD4+ T cells and an upregulation of CD8+ T cells in DFUNH, although not statistically significant (S5A–S5D Fig). This suggests that the depletion of CD8+ T cells may be beneficial for the healing of diabetic wounds. Plasma cells are abundant in DFUs non-healing wounds, indicating a possible association with B lymphocyte differentiation and non-healing wounds. Generally, M1 macrophages promote inflammatory responses, while M2 macrophages primarily inhibit inflammation and promote angiogenesis. Our results showed an increase in the proportion of M2 cells in DFUNH, while a decrease in proportion was observed in DFUH, consistent with the study by Theocharidis et al. (Fig 2C) [7]. Compared to the DS group, the M1/M2 ratio decreased in the DFUNH group, and compared to the DFUNH group, this ratio significantly increased in the DFUH group (Fig 2F). Regarding monocytes in the blood, the classical phenotype (CD14+ monocytes) is similar to the pro-inflammatory phenotype described earlier, while the non-classical phenotype (CD16+ monocytes) is similar to anti-inflammatory monocytes [23]. Similar to macrophages, the CD14/CD16 ratio decreased in the DFUNH group compared to the DS group, and this ratio significantly increased in the DFUH group compared to the DFUNH group (Fig 2E). Furthermore, a lower negative regulation score for local inflammation response was observed in DFUH wound sites, while a higher negative regulation score for systemic inflammation response was observed in peripheral blood, indicating local inflammatory activation and systemic inflammation suppression in healing wounds (Fig 2G and 2H). These results suggest that a moderate local inflammatory response is necessary for the healing of diabetic wounds.

### 3. Cross-cell expression changes of reversed non-healing associated genes in healing wounds

**Fig 3:**
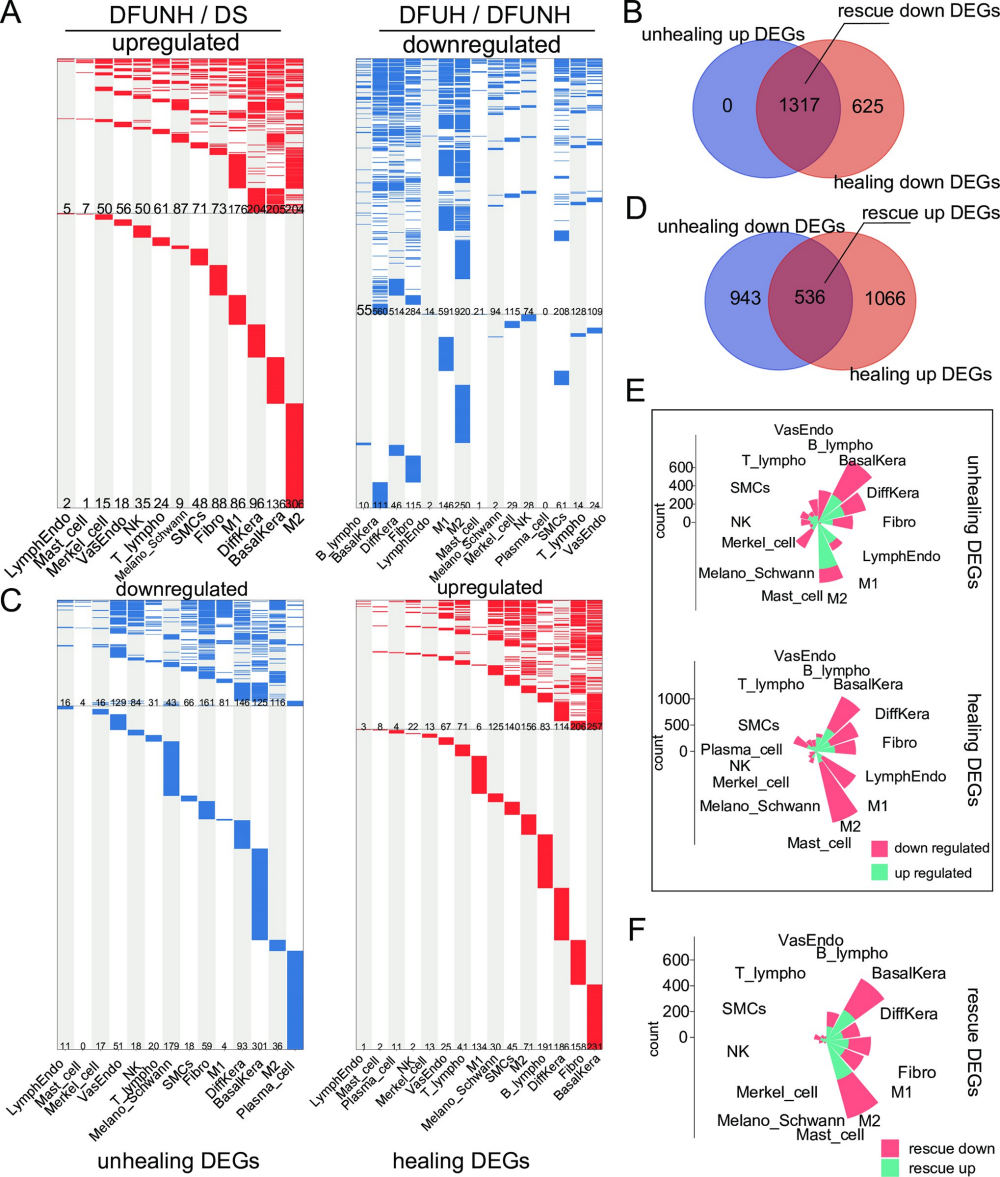
Screening of unhealing genes, healing genes, and rescue genes in skin. (A, C) Heatmaps display the distribution of DEGs in the skin across different cell types. Each column represents a cell type, and each row represents a gene. Red indicates genes that are upregulated in DFUNH compared to DS, as well as genes that are upregulated in DFUH compared to DFUNH (LogFC > 0.5, adjusted p-value < 0.05). Blue indicates genes that are downregulated in DFUNH compared to DS, as well as genes that are downregulated in DFUH compared to DFUNH (LogFC < -0.5, adjusted p-value < 0.05). Gray indicates no significant change (|LogFC| < 0.5). Unhealing DEGs refer to genes that show changes in the DFUNH group compared to the DS group, while healing DEGs refer to genes that show changes in the DFUH group compared to the DFUNH group. Rescue DEGs are genes that exhibit opposite changes in expression compared to unhealing DEGs within healing DEGs. (B, D) Venn diagrams display the number of unhealing, healing, and rescue DEGs. The overlapping regions represent the number of downregulated rescue DEGs (B) and upregulated rescue DEGs (D). (E, F) The rose plots display the number and cell type distribution of unhealing DEGs, healing DEGs (E), and rescue DEGs (F) in the skin.

**Fig 4:**
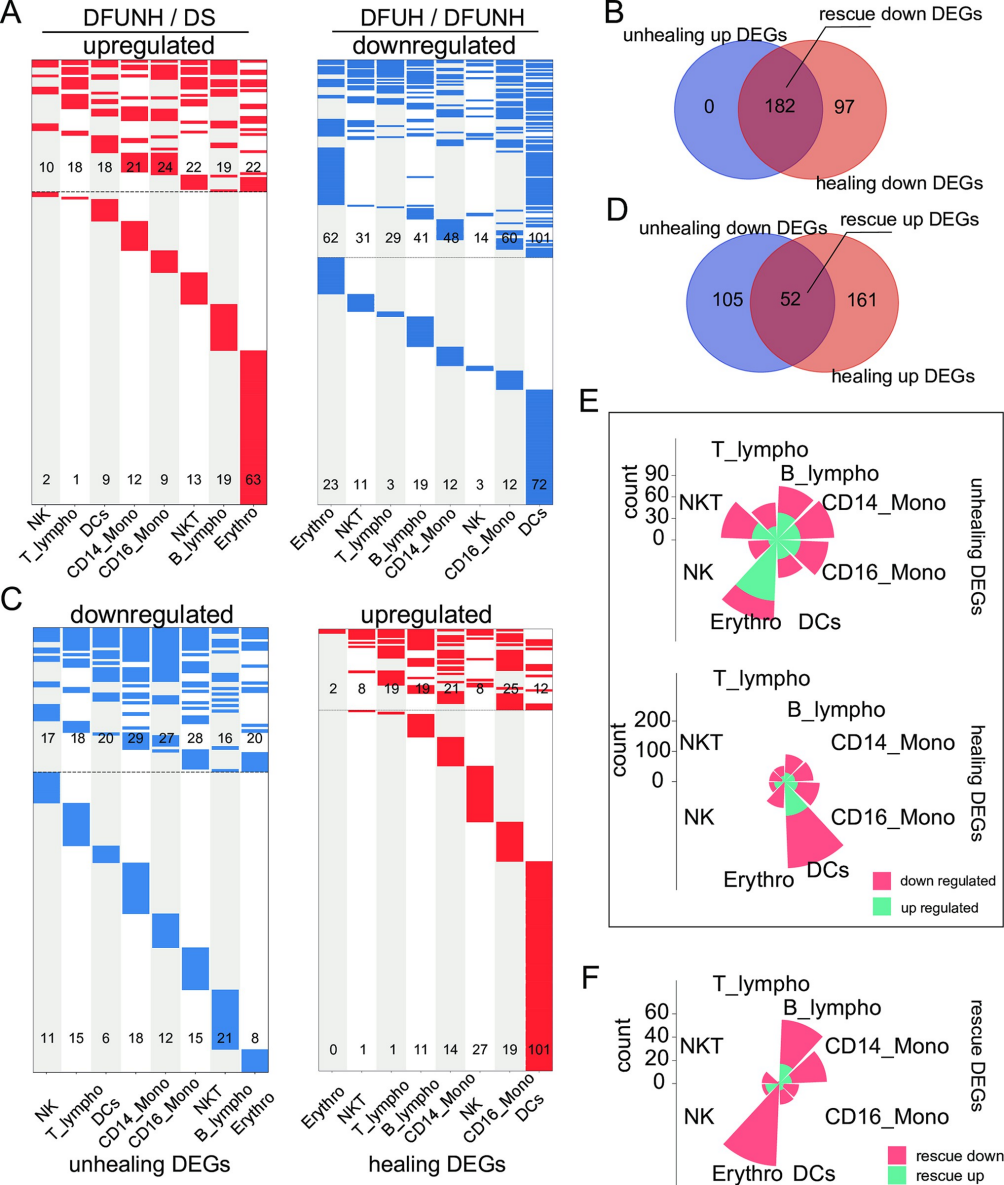
Screening of unhealing genes, healing genes, and rescue genes in blood. (A, C) Heatmaps display the distribution of DEGs in the peripheral blood across different cell types. Each column represents a cell type, and each row represents a gene. Red indicates genes that are upregulated in DFUNH compared to DS, as well as genes that are upregulated in DFUH compared to DFUNH (LogFC > 0.5, adjusted p-value < 0.05). Blue indicates genes that are downregulated in DFUNH compared to DS, as well as genes that are downregulated in DFUH compared to DFUNH (LogFC < -0.5, adjusted p-value < 0.05). Gray indicates no significant change (|LogFC| < 0.5). Unhealing DEGs refer to genes that show changes in the DFUNH group compared to the DS group, while healing DEGs refer to genes that show changes in the DFUH group compared to the DFUNH group. Rescue DEGs are genes that exhibit opposite changes in expression compared to unhealing DEGs within healing DEGs. (B, D) Venn diagrams display the number of unhealing, healing, and rescue DEGs. The overlapping regions represent the number of downregulated rescue DEGs (B) and upregulated rescue DEGs (D). (E, F) The rose plots display the number and cell type distribution of unhealing DEGs, healing DEGs (E), and rescue DEGs (F) in the peripheral blood.

In order to elucidate the molecular events associated with healing and non-healing, we identified thousands of DEGs between DFUNH and DS, as well as between DFUH and DFUNH, which we referred to as "non-healing-related DEGs" and "healing-related DEGs," respectively. Through further comprehensive comparative analysis of these differentially expressed genes (DEGs), key DEGs that exhibited opposite expression trends in healing wounds and non-healing wounds were identified, and therefore referred to as "rescue DEGs" (Figs 3A–3D and 4A–4D; see Methods section for details).

Next, to distinguish the distribution of non-healing DEGs and healing DEGs in each cell type, we attributed the non-healing, healing, and rescue DEGs to each cell type. Clearly, as shown in the rose plot and cell-gene network plot, non-healing-related and healing-related genes are predominantly distributed in keratinocytes (both differentiated and undifferentiated), fibroblasts, M1, and M2 macrophages in the skin tissue (Fig 3E and S6A and S6B Fig). Similarly, rescue DEGs are also primarily distributed in these cell types (Fig 3F and S6C Fig). This suggests that keratinocytes, fibroblasts, M1, and M2 are the main responsive cell types in the wound healing process and play a significant role. In peripheral blood, non-healing DEGs were mainly distributed in erythrocytes, B cells, NKT cells, CD14, and CD16 cells, while healing-related DEGs were mainly distributed in DCs (Fig 4E and S6D and S6E Fig). Rescue DEGs were primarily distributed in B cells, CD14 monocytes, and erythrocytes (Fig 4F and S6F Fig). This indicates that B cells, CD14 monocytes, and erythrocytes are the main blood cell types involved in the response during wound healing. These analyses highlight the cell specificity of unhealing and healing DEG distributions and suggest that a multi-cell type comparison may provide a more comprehensive portrait for a better understanding of the mechanisms underlying unhealing and healing.

**Fig 5:**
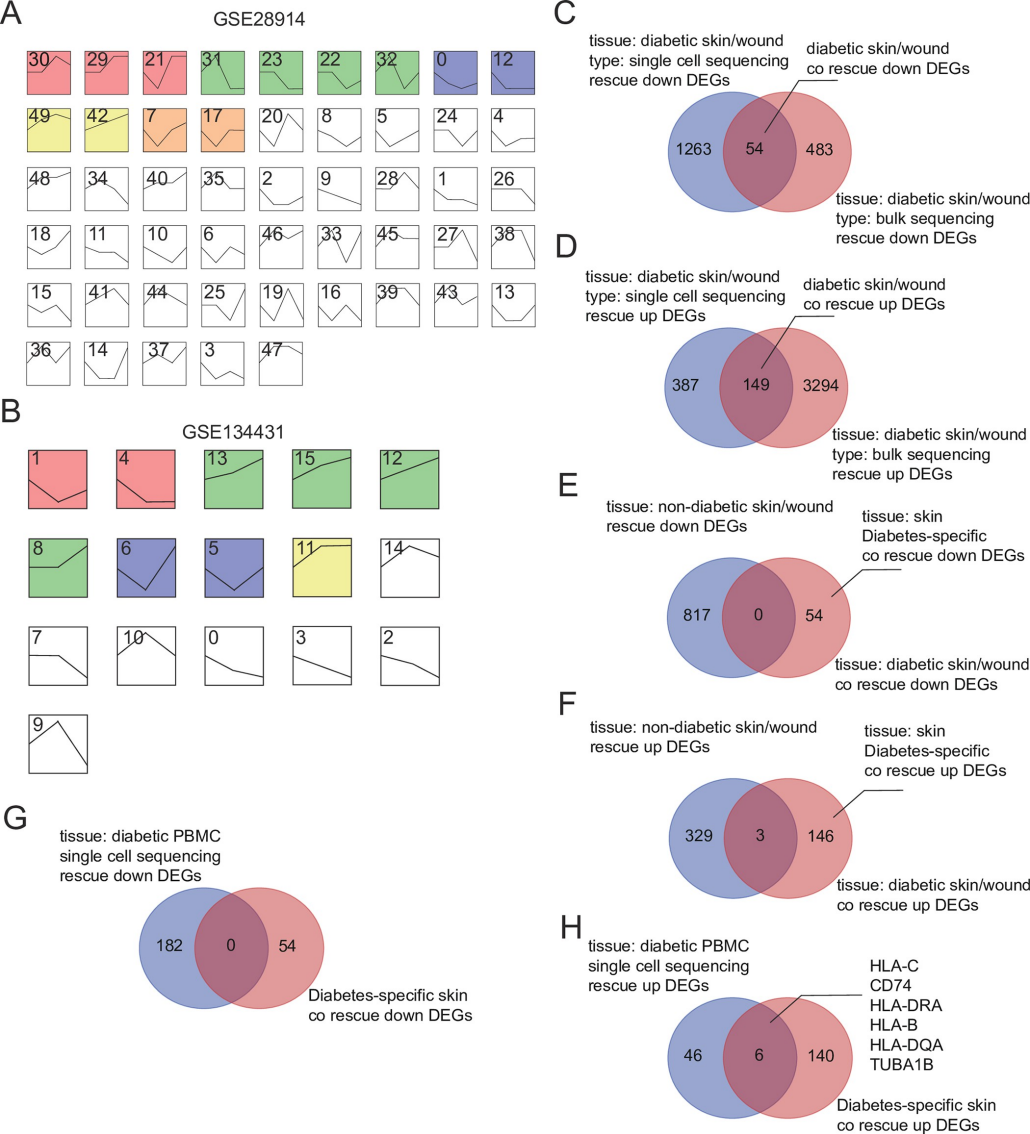
Identification of genes exhibiting specific changes during the wound healing process. (A) Time kinetics of gene expression during acute non-diabetic wound healing. The black line represents the overall trend in each profile, and color-coded profiles indicate statistically significant differences (P < 0.05). Profiles of the same color represent clusters with similar expression trends. (B) Time kinetics of gene expression during diabetic wound healing. The black line represents the overall trend in each profile, and color-coded profiles indicate statistically significant differences (P < 0.05). Profiles of the same color represent clusters with similar expression trends. (C-D) Common rescue down (C) or rescue up (D) genes in bulk transcriptome (GSE134431) and single-cell transcriptome (GSE165816) of diabetic wounds. (E-F) Intersection of rescue down or rescue up genes common to diabetic wounds and rescue down/up genes during acute wound healing, to exclude intersecting genes and obtain uniquely expressed rescue down (E) or rescue up (F) genes during diabetic wound healing. (G-H) Venn diagram showing the number of shared upregulated (H) or downregulated (G) rescue DEGs in the skin and peripheral blood.

To further narrow down the scope of DEGs and exclude genes with similar trends of changes during acute wound healing, we performed a combined analysis of the selected DEGs with the bulk transcriptome datasets GSE134431 (diabetes wound-related) and GSE28914 (acute wound healing-related). Firstly, using STEM software, we obtained the rescue up and rescue down genes in GSE134431 and GSE28914, respectively (see Methods). Specifically, genes that followed an increasing and then decreasing trend in expression throughout the entire wound healing process were referred to as rescue down genes, while genes that exhibited a decreasing and then increasing trend were referred to as rescue up genes. As shown in Fig 5A, genes contained in clusters 30, 31, 32, and 49 of the GSE28914 dataset were rescue down genes, while clusters 21, 22, 7, and 17 contained rescue up genes. Similarly, in the GSE134431 dataset, clusters 1, 6, and 5 contained rescue up genes, while clusters 14, 10, and 9 contained rescue down genes (Fig 5B). First, we took the intersection of rescue up and rescue down genes in the skin tissue from GSE165816 and rescue up and rescue down genes in GSE134431, resulting in 54 common rescue down genes and 149 common rescue up genes (Fig 5C and 5D). Then, we took the intersection of the aforementioned common genes with the rescue down and rescue up genes during acute wound healing to exclude genes that showed changes in the acute wound healing process, resulting in 54 unique rescue down genes and 146 unique rescue up genes in diabetes wounds (Fig 5E and 5F). The changes in these genes were considered specific to the diabetes wound healing process.

**Fig 6:**
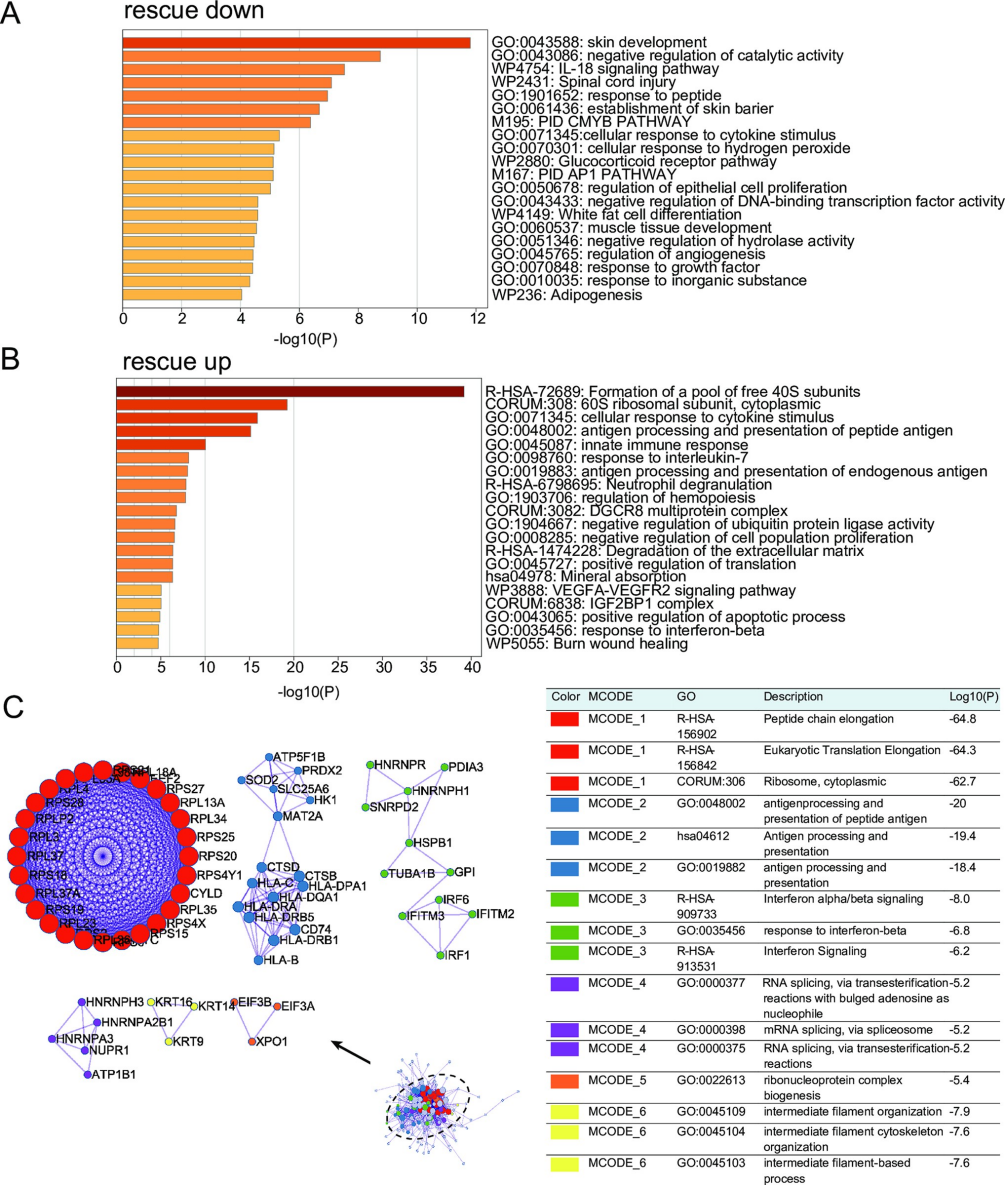
The enrichment analysis of diabetes-specific rescue genes. (A-B) GO enrichment analysis of the aforementioned uniquely expressed rescue down (A) and rescue up (B) genes. (C) PPI network constructed by the common rescue down and rescue up genes and the core module within the network. Different colors represent different core modules.

Next, we explored the biological significance of these unique rescue DEGs in diabetes wounds using Gene Ontology (GO) and pathway analysis (http://metascape.org/). For rescue down DEGs, enrichment was observed in categories such as skin development, IL-18 signaling pathway, establishment of skin barrier, PIDCMYB PATHWAY, cellular response to cytokine stimulus, cellular response to hydrogen peroxide, Glucocorticoid receptor pathway, regulation of epithelial cell proliferation, muscle tissue development, regulation of angiogenesis, and response to growth factor (Fig 6A). For rescue up DEGs, enrichment was observed in categories such as cellular response to cytokine stimulus, antigen processing and presentation of peptide antigen, innate immune response, response to interleukin-7, antigen processing and presentation of endogenous antigen, neutrophil degranulation, regulation of hematopoiesis, negative regulation of ubiquitin protein ligase activity, negative regulation of cell population proliferation, degradation of the extracellular matrix, positive regulation of translation, VEGFA-VEGFR2 signaling pathway, positive regulation of apoptotic process, response to interferon-beta, and burn (Fig 6B). Further PPI analysis revealed that the core network functions constructed by these rescue down and rescue up genes were mainly associated with antigen processing and presentation, interferon signaling, and intermediate filament organization (Fig 6C). The gene expression scores of the GO terms "antigen processing and presentation" and "response to interferon beta" in the three clinical groups were further determined using the AddModuleScore function, and the results were consistent with the above findings (S7A and S7B Fig). Antigen-presenting cells, such as macrophages and dendritic cells, present and process antigens from the wound environment and migrate to lymph nodes, where they present the processed antigens to T cells. This leads to the activation of specific immune responses against the antigens and helps clear pathogens or abnormal cells present in the wound [24]. Impairment of antigen processing and presentation in DFUNH may result in clearance defects of pathogens and other antigens, leading to the sustained non-healing state of the wound. Interferon signaling plays an important regulatory role in antigen presentation and immune response by regulating the activation, maturation, and MHC molecule expression of APC cells, as well as directly affecting the functions of other immune cells [25, 26]. Studies have shown that the absence of IFN-γ and IFN-κ impairs wound healing in diabetes wounds [27, 28]. To identify the shared rescue DEGs between skin tissue and peripheral blood, we created Venn diagrams of rescue DEGs in different tissues. The results showed that six downregulated unhealing-related genes (HLA-C, CD74, HLA-DRA, HLA-B, HLA-DQA, TUBA1B) were reactivated in healing wounds in both skin and peripheral blood (Fig 5G and 5H). These DEGs were downregulated in non-healing wounds and restored in healing wounds. HLA-B and HLA-C belong to the MHC class I molecules, while HLA-DRA and HLA-DQA belong to the MHC class II molecules. HLA-II molecules can enhance the binding and presentation capacity of antigen-presenting lymphocytes, mediating the actions of other immune components [29]. These molecules also act as co-stimulatory molecules, enhancing inflammation and mediating the production of cytokines such as interferons [30], which are associated with increased collagen deposition [31]. The systemic (in blood) downregulation of the above-mentioned HLA molecules may be one of the reasons for impaired antigen processing and presentation in DFUNH. In addition, we evaluated the potential diagnostic value of these 6 genes in the bulk dataset GSE134431. Among them, CD74, HLA-B, and HLA-DRA showed AUC values greater than 0.7, indicating that these three genes have the potential to be biomarkers for non-healing DFUs (S8 Fig).

### 4. Disrupted ECM signaling in non-healing diabetic foot ulcers

**Fig 7:**
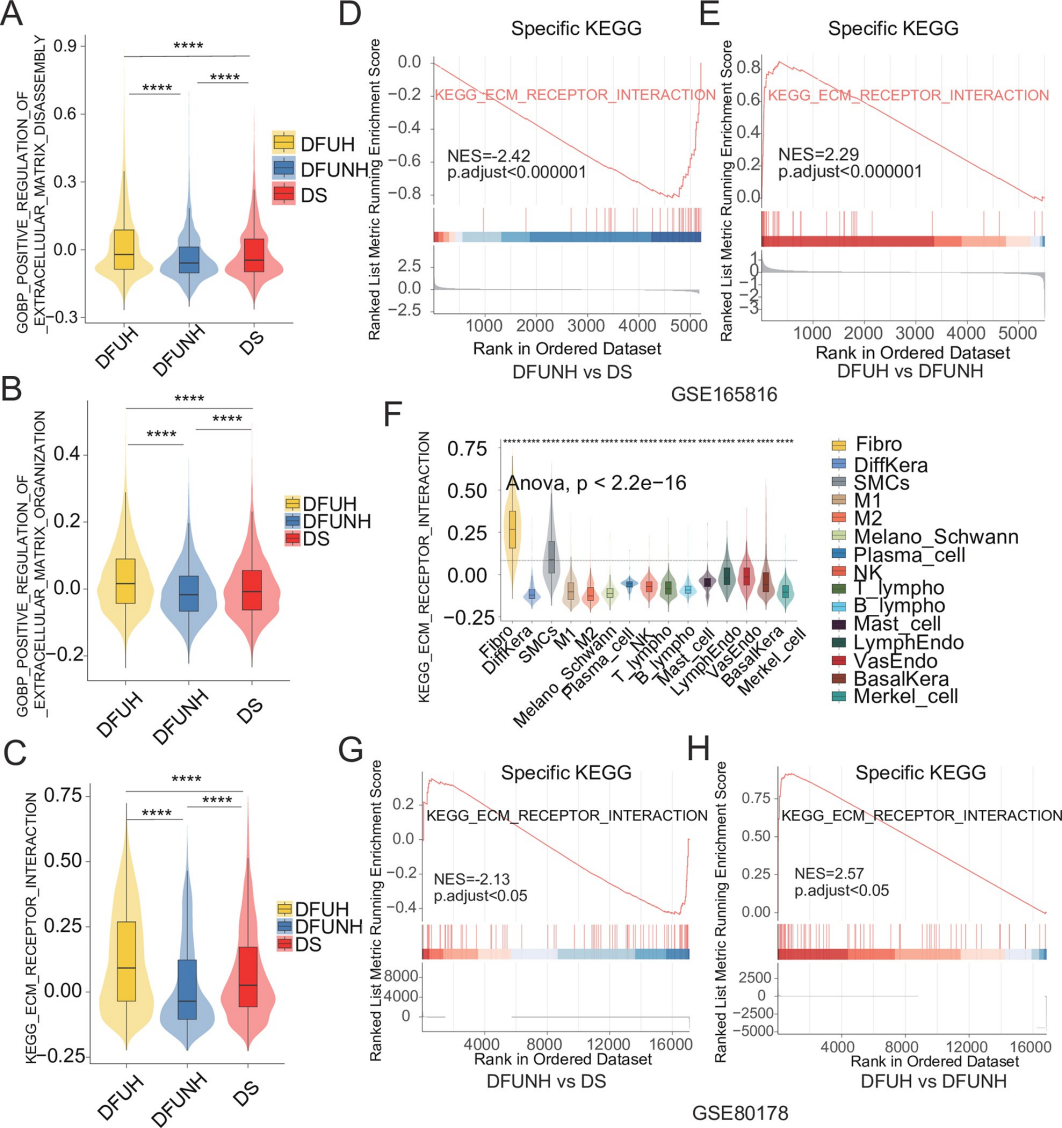
Activity of ECM assembly and degradation, as well as ECM-receptor signaling, in the skin tissues of three clinical groups. (A) Gene expression scores of ECM degradation signals in the single-cell datasets DS, DFUNH, and DFUH groups. ****, P<0.05. (B) Gene expression scores of ECM assembly signals in the single-cell datasets DS, DFUNH, and DFUH groups. ****, P<0.05. (C) Gene expression scores of ECM-receptor interaction signals in the single-cell datasets DS, DFUNH, and DFUH groups. ****, P<0.05. (D) Enrichment results of ECM-receptor interaction signals in DFUNH compared to DS. Adjusted p <0.05, |NES|> = 1. Data source: GSE165816. (E) Enrichment results of ECM-receptor interaction signals in DFUH compared to DFUNH. Adjusted p <0.05, |NES|> = 1. Data source: GSE165816. (F) Gene expression scores of ECM-receptor interaction signals in various skin cell types. Data source: GSE165816. The dashed line represents the baseline level of base mean. (G) Enrichment results of ECM-receptor interaction signals in DFUNH compared to DS. Adjusted p <0.05, |NES|> = 1. Data source: GSE134431. (H) Enrichment results of ECM-receptor interaction signals in DFUH compared to DFUNH. Adjusted p <0.05, |NES|> = 1. Data source: GSE134431.

The extracellular matrix (ECM) is a complex network of macromolecules secreted by cells into the extracellular space, accounting for more than one-third of the body’s mass. ECM components not only provide dynamic tissue integrity but also serve as signaling molecules involved in driving numerous biological reactions, making their dysregulation a direct or indirect cause of most chronic diseases [32]. From an immunological perspective, the ECM also includes various secreted proteins, including cytokines, chemokines, and growth factors, which potentially participate in immune cell regulation [33]. The results presented herein demonstrate a decrease in the proportion of fibroblasts, the primary cell type responsible for synthesizing and secreting ECM major components, in non-healing diabetic foot ulcers (DFUNH). To further investigate the differential ECM-related signaling activities in DS, DFUNH, and DFUH group, we utilized the AddModuleScore function to evaluate the gene expression scores of ECM-receptor signaling pathways among different clinical groups. The results showed elevated ECM degradation and assembly activities in both the DFUH and DS groups compared to the DFUNH group, with higher ECM-receptor signaling pathway scores observed in the DFUH and DS groups compared to the DFUNH group (Fig 7A–7C). These findings suggest an inactivation of the ECM-receptor signaling pathway in DFUNH. Moreover, the enrichment analysis of GSE165816 and bulk transcriptome data confirmed the downregulation of ECM-receptor signaling levels in DFUNH (Fig 7D, 7E, 7G and 7H). Additionally, fibroblasts were identified as the cell type with the highest ECM receptor signaling score (Fig 7F), indicating their dominant role in ECM-receptor signaling.

**Fig 8:**
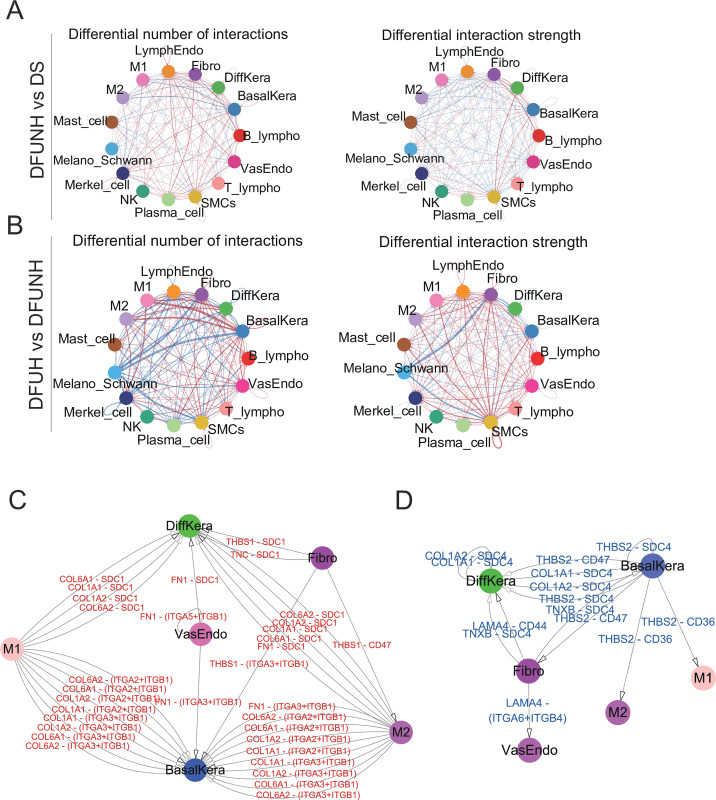
Changes in ligand-receptor interactions in ECM-receptor signaling between different cell types between the DS and DFUNH groups, as well as between the DFUNH and DFUH groups. (A-B) Network diagrams depict the changes in ligand-receptor interactions between different cell types in the skin DFUNH/DS (A) and DFUH/DFUNH (B) comparison groups. Communication between cells is represented by connecting lines. The thickness of the lines is positively correlated with the number or intensity of ligand-receptor interaction events. Red or blue color indicates an increase or decrease in signaling in the second dataset (DFUNH or DFUH) compared to the first dataset (DS or DFUNH). (C-D) Extracted ligand-receptor pairs included in Fibro, BasalKera, DiffKera, M1, M2, and VasEndo cells for "rescue up" (C) and "rescue down" (D). The starting point of an arrow represents the cell containing the ligand, while the endpoint of the arrow points to the cell type containing the receptor targeted by that ligand. "Rescue up" and "rescue down" are indicated by red and blue arrows, respectively.

To further explore the molecular events associated with ECM-related signaling during the wound healing process in diabetic ulcers, the distribution of ECM-receptor signaling molecules in the skin tissues of DS, DFUNH, and DFUH groups was analyzed using Cellchat. The cell-cell communication network depicted by the computational analysis revealed enhanced or diminished ECM-receptor signaling between DS and DFUNH, as well as between DFUNH and DFUH (Fig 8A and 8B, S9A and S9B Fig). Similarly, rescue up and rescue down ligand-receptor pairs were obtained using a similar approach for ECM-receptor signaling. Rescue up ligand-receptor pairs refer to those that are downregulated in DFUNH (compared to DS) and upregulated in DFUH (compared to DFUNH). Rescue down ligand-receptor pairs refer to those that are upregulated in DFUNH (compared to DS) and downregulated in DFUH (compared to DFUNH) (see methods for details). Cell communication analysis revealed a significant reduction in COLLAGEN signaling in DFUNH tissues compared to DS, primarily involving COL1 and COL6 ligands and their corresponding receptors, along with diminished FN1, THBS, and TENASCIN signaling. These signals were largely restored in DFUH (S3 Table). Collagen is a major structural component of the extracellular matrix, providing support and structure to various tissues in the body [34]. Studies have shown that the effective domains of recombinant human COL1A1-collagen can induce cell proliferation and collagen synthesis in human dermal fibroblasts, as well as enhance cell migration and elastin production. Peptides derived from human COL1A2 have also been shown to promote wound healing and elastin production. Human collagen I alpha-2-derived peptide can improve the synthesis of type I collagen, cell proliferation, cell migration, and elastin synthesis [35]. Additionally, fragments of type I collagen can serve as potent chemotactic agents for neutrophils, enhance phagocytosis and immune response, and regulate gene expression, making them inflammatory mediators [36, 37]. They can also effectively stimulate angiogenesis in vitro and in vivo through the involvement of specific integrin receptors [38]. Type VI collagen, a non-fibrillar collagen, is expressed in many connective tissues and participates in the formation of the ECM. It interacts with various key ECM components, including type I and type II fibrillar collagens, basement membrane type IV collagens, fibronectin, and others. In connective tissue ECM, type VI collagen forms highly branched fibrillar networks surrounding the main fibrous collagens I, II, and III. Additionally, through its interaction with basement membrane type IV collagen, it anchors blood vessels, nerves, and mesenchymal cells. It also serves as a reservoir for platelet-derived growth factors, keratinocyte growth factor, matrix metalloproteinases-1, -2, -3, -8, and -9, IL-2, and cytokine thymic stromal lymphopoietin, regulating their activity and availability [39]. Type VI collagen acts as an early sensor for injury/repair responses and can regulate fibrogenesis by modulating cell-cell interactions, stimulating mesenchymal cell proliferation, and preventing apoptosis [40].

It is worth noting that the restored COL1A1, COL1A2, COL6A1, and COL6A2 in DFUH mainly originate from M1 and M2 cells, while their corresponding receptors ITGA2, ITGB1, ITGA3, ITGA9, SDC1, and SDC4 are predominantly present in BasalKera and DiffKera cells (Fig 8C and S3 Table). This suggests that weakened cell communication between the COL1 and COL6 ligands derived from M1 and M2 cells and their corresponding receptors from BasalKera and DiffKera cells may be an important factor contributing to the non-healing of wounds. However, there is also a small subset of COLLAGEN signaling (mainly COL1 and COL6) between specific cell types that increases in DFUNH and decreases in DFUH (rescue down) (Fig 8D and S4 Table). Since the majority of COLLAGEN signaling is diminished in DFUNH, these increased COLLAGEN signals in DFUNH can be disregarded.

Furthermore, we observed an imbalance in THBS signaling in DFUNH. Specifically, THBS1 is mainly present in the rescue up signal, while THBS2 is mainly present in the rescue down signal. In the rescue up signal, the THBS1 ligand, derived primarily from Fibro, corresponds to receptors from various cell types such as BasalKera, LymphoEndo, M2, DiffKera, and Merkel cells (S3 Table). In the rescue down signal, the THBS2 ligand is predominantly derived from BasalKera, and its corresponding receptors are distributed in M1, M2, Fibro, and DiffKera. THBS1 and THBS2 seem to play distinct and non-overlapping roles in skin wound healing. Studies have shown that THBS1 knockout mice exhibit delayed wound healing, prolonged inflammation, and reduced TGF-β in the wound bed [41, 42]. This indicates the positive role of THBS1 in wound healing. In contrast, THBS2 knockout mice show accelerated full-thickness wound healing, enhanced angiogenesis, and changes in the remodeling phase of healing [43, 44]. This suggests the negative role of THBS2 in wound healing. Additionally, LAMININ signaling is only present in the rescue down signal and absent in the rescue up signal. The LAMA4 ligand mainly originates from Fibro, while its corresponding receptors ITGA6, ITGB1, ITGB4 are located in LymphoEndo and VasEndo. Similarly, FN1 signaling is exclusively found in the rescue up signal. The FN1 ligand is primarily derived from M2 and VasEndo, and its corresponding receptors ITGA3, ITGA5, ITGB1, SDC1, and SDC4 are distributed in BasalKera, VasEndo, DiffKera, and Merkel cells. FN1 plays a crucial role in wound healing by promoting cell adhesion, migration, angiogenesis, and providing a scaffold for tissue regeneration [45–47]. Its interaction with various cell types and growth factors helps coordinate effective repair of damaged tissue. The loss of FN1 signaling in DFUNH may be an important factor contributing to the non-healing of wounds [48–50].

The ligand-receptor pairs TNXB-SDC4 are upregulated in DFUNH and downregulated in DFUH, while the pairs TNC-SDC1 and TNC-SDC4 are downregulated in DFUNH and upregulated in DFUH. This suggests that TNC and TNXB may play different biological roles in DFUNH. During the inflammatory phase, TNC can induce the synthesis of pro-inflammatory cytokines through TLR4. In the proliferative phase, the significant increase in TNC promotes angiogenesis [51]. Similarly, we evaluated the potential diagnostic value of COL1A1, COL1A2, COL6A1, COL6A2, FN1, THBS1, THBS2, TNC, and TNXB genes in the bulk dataset GSE134431. Among them, COL6A1, COL6A2, and FN1 showed AUC values greater than 0.7, indicating that these three genes also have the potential to be biomarkers for non-healing DFUs (S8 Fig).

### 5. MMPs and TIMPs are widely downregulated in DFUNH

**Fig 9:**
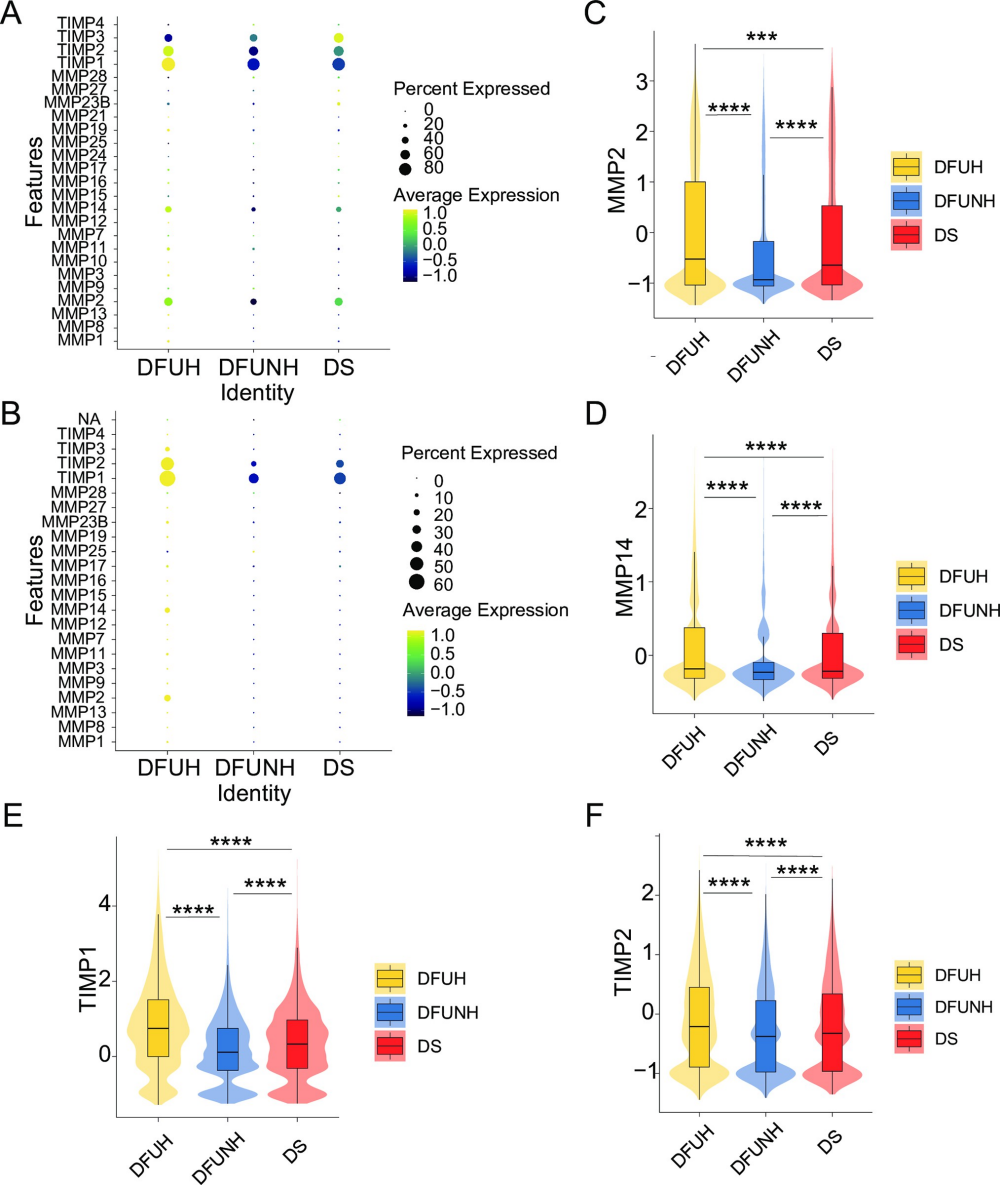
Expression patterns of MMPs and TIMPs in three clinical groups. (A-B) Dot plots depicting the expression of MMP and TIMP gene families in the skin (A) and peripheral blood (B) of the three clinical groups. The size of the dots represents the percentage of gene expression in different clinical groups, while the color represents the average expression level of the genes; dark blue: low, yellow: high. (C-F) Overall expression patterns of MMP2, MMP14, TIMP1, and TIMP2 in the skin tissue of the three clinical groups. ***, P < 0.001; ****, P < 0.0001.

Matrix metalloproteinases (MMPs) are a family of zinc-dependent extracellular matrix (ECM) remodeling enzymes that can degrade almost all components of the ECM. Changes in the expression of MMPs lead to abnormal degradation of the ECM, which is the initial cause of chronic degenerative diseases and the development of vascular complications in diabetes. Tissue inhibitors of matrix metalloproteinases (TIMPs) within the ECM inhibit the proteolytic activity of MMPs. TIMPs are important regulatory factors in ECM metabolism, tissue remodeling, and cellular behavior. Therefore, similar to MMPs, TIMPs regulate angiogenesis, cell proliferation, and cell apoptosis. The regulatory roles of MMPs and TIMPs can affect the interaction and signaling between cells and the ECM, thereby regulating the activity and effects of ECM receptor signaling pathways [52, 53]. If the strict balance between MMP activity and inhibition is disrupted, wounds can progress to a state of increased ECM degradation, altered cytokine profiles, and degradation of growth factors, ultimately leading to delayed or impaired wound closure [54, 55]. We analyzed the expression of 21 human MMPs and 4 TIMPs in DFUs skin and blood. As shown in Fig 9, the expression levels of MMP2, MMP14, TIMP1, and TIMP2 were decreased in the skin and blood of the DFUNH group compared to the DS group, while their expression was restored in the DFUH group compared to the DFUNH group (Fig 9A–9F). MMP2 and MMP14 are mainly expressed in Fibro (S10C and S10D Fig), TIMP1 is mainly expressed in Fibro, LymphoEndo, M1, M2, Mast cell, and SMCs, and TIMP2 is mainly expressed in Fibro, LymphoEndo, M1, M2, Mast cell, Melano_schwann, and SMCs (S10A and S10B Fig). In fibroblasts, TIMP1 and MMP14 were downregulated in DFUNH compared to DS, while their expression was restored in DFUH compared to DFUNH (S10A and S10D Fig). TIMP2 and MMP2 were both highly expressed in DFUNH and DFUH compared to the DS group, but their expression was higher in DFUH (S10B and S10C Fig).

Studies have shown that MMP2 accelerates cell migration. MMP2 regulates angiogenesis in the wound healing process by activating pro-angiogenic cytokines, including TNF-α and VEGF, and by producing anti-angiogenic peptides, such as endostatin derived from type XVII collagen, expressed in the basement membrane [56, 57]. However, there are also studies suggesting that the activity of MMP2 is several-fold higher in non-healing wounds compared to healing wounds, and inhibiting MMP2 contributes to promoting the healing process [58]. MMP14 activates MMP-2 by degrading pro-MMP-2 [59]. The absence of MMP14 results in defective turnover of type I collagen and impaired activation of MMP-2 [60, 61]. In addition, MMP14 regulates epithelial cell proliferation in the wound healing process by altering the expression of KGF receptors [62]. Besides controlling proliferation, MMP14 also promotes epithelial cell migration in vitro by cleaving syndecan-1, CD44, and laminin-332 [63–65]. MMP14 knockout mice exhibit early onset and increased mortality rates [66]. TIMP-1 is present in the epithelial cells of healing excision and burn wounds, and it is also expressed in wound fibroblasts, particularly in fibroblasts near human blood vessels [67, 68]. Evidence suggests that skin injuries in diabetes lead to increased production of TIMP-1 and type I and III procollagen in diabetic skin animal models. Proper levels of active TIMP-1 protein can effectively protect cells treated with AGEs from apoptosis. Local administration of active TIMP-1 protein or TIMP-1 gene therapy at the wound site can be used as a strategy to accelerate diabetic wound healing [69]. TIMP-1 can inhibit the activity of MMP-9 [70]. TIMP-1 may participate in epidermal regeneration by stabilizing the basement membrane zone and regulating matrix remodeling and wound bed angiogenesis. A biopsy of chronic diabetic skin ulcer wound tissue showed decreased expression of TIMP-2 [71]. The lack of TIMP-2 near the migrating epithelial wound edge may lead to uncontrolled activity of MMP-2 in chronic ulcers. TIMP-2 has been shown to impair [65] or accelerate [72] cell migration in vitro. In fact, recombinant human TIMP-2 (rh-TIMP-2) accelerates wound closure in diabetic mice, and GPI anchor modification of TIMP-1 (TIMP-1-GPI) increases the rate of healing in a excision wound model in humans [72]. In hypoxic human keratinocytes, secretion of MMP-2, MMP-9, and TIMP-2 is reduced, while in human monocytes, MMP-9 reduction and TIMP-1 increase are observed [73, 74]. Our results imply a protective role of MMP2, MMP14, TIMP1, and TIMP2 in wound healing.

### 6. Healing-related fibroblast subtypes drive DFUs healing through promoting matrix remodeling

**Fig 10:**
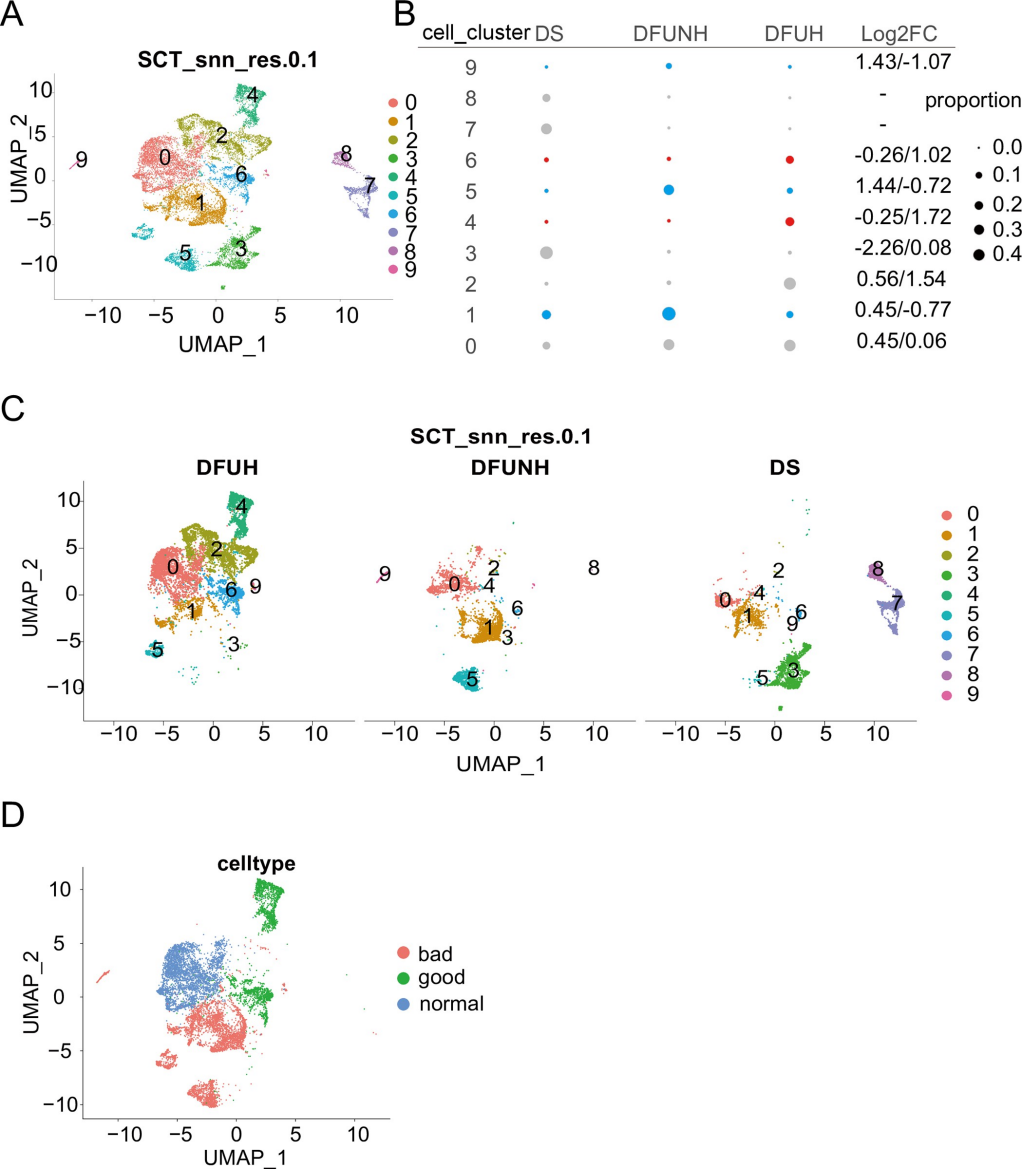
Identification and characterization of fibroblast subpopulations with specific gene features associated with healing DFUs. (A) UMAP analysis describes 10 fibroblast subpopulations at a resolution of 0.1. (B) Relative changes in cell proportions across the three groups of subpopulations. The numbers on the right represent Log2FC values of cell proportions (DFUNH/DS and DFUH/DFUNH). The cell subpopulation types highlighted in red are downregulated during the unhealing process and upregulated during the healing process, while the cell subpopulation types highlighted in blue are upregulated during the unhealing process and downregulated during the healing process. Gray indicates cell subpopulation types with no significant proportional changes. (C) UMAP dimensionality reduction plot of cell subpopulation distribution in the DS, DFUNH, and DFUH groups. (D) Cluster 0 classified as "normal" cells with typical skin fibroblast functions, clusters 4 and 6 classified as "good" cells, and clusters 1, 5, and 9 classified as "bad" cells.

**Fig 11:**
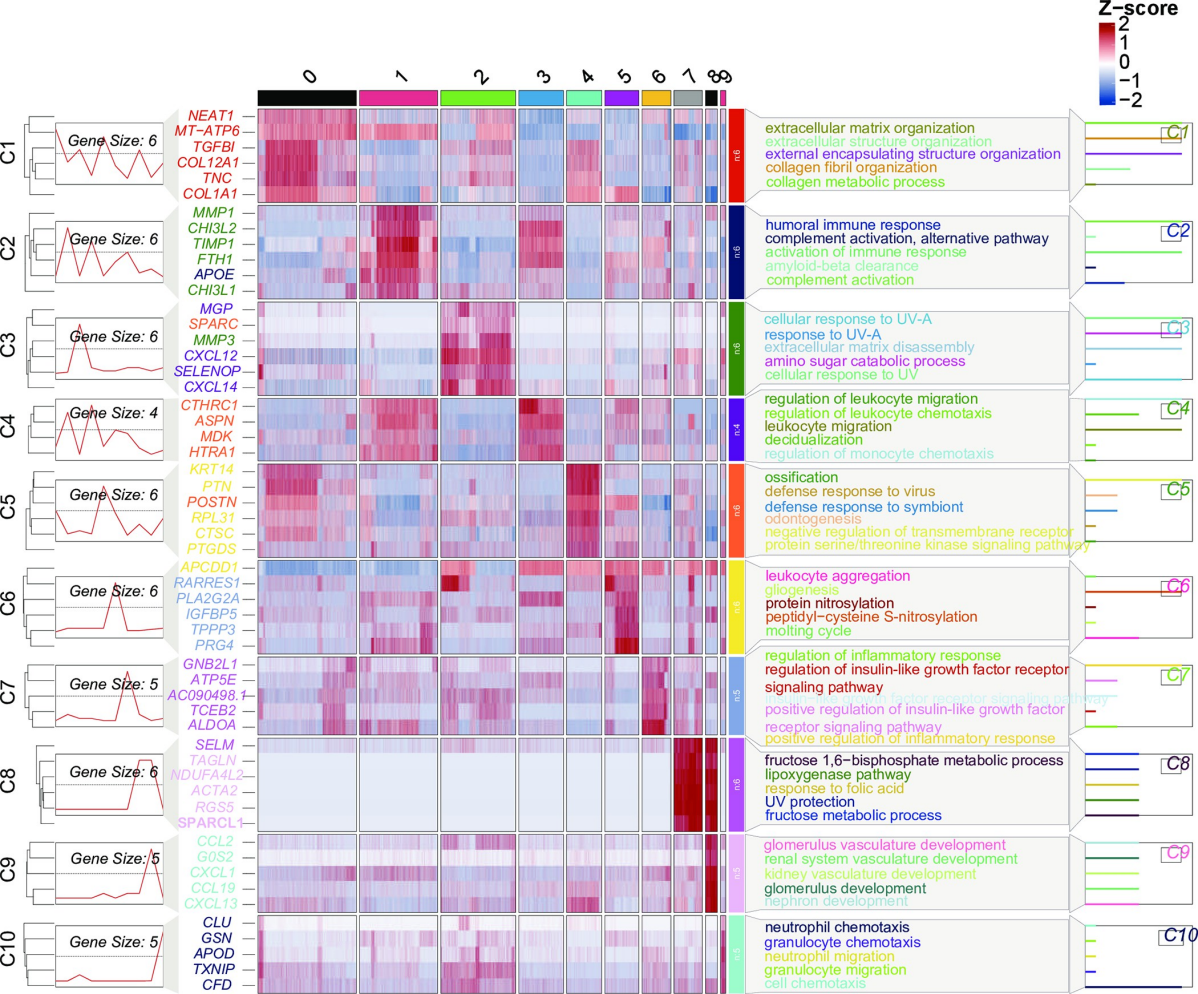
Heatmap showing the highest expression genes in each subgroup and their GO enrichment analysis results.

To further characterize the role of fibroblasts in wound healing, we conducted a detailed analysis of fibroblasts, resulting in the identification of 10 subclusters (Fig 10A), representing distinct molecular states or fibroblast subtypes. Most subclusters exhibited distinct expression profiles, indicating the heterogeneity of the fibroblast population (Fig 11). To further explore the heterogeneity of each subcluster, we performed gene ontology (GO) enrichment analysis for the top 6 genes in each subcluster. The results showed that Cluster 0 represented classical fibroblast types, with enrichment in processes such as extracellular matrix organization, extracellular structure organization, external encapsulating structure organization, collagen fibril organization, and collagen metabolic process (Fig 11). Cluster 1 was associated with complement activation and immune response. Cluster 2 was related to response to UV and ECM degradation. Cluster 3 was associated with leukocyte migration and chemotaxis. Cluster 4 was related to defense response to viruses and symbionts. Cluster 5 was associated with leukocyte aggregation. Cluster 6 was related to regulation of immune response and insulin-like growth factor receptor signaling pathway. Cluster 7 was related to fructose 1,6-bisphosphate metabolic process, lipoxygenase pathway, response to folic acid, UV protection, and fructose metabolic process, indicating its involvement in metabolic regulation. Cluster 8 was associated with kidney development, and Cluster 9 was related to granulocyte/neutrophil migration and chemotaxis (Fig 11).

We compared the proportions of fibroblast subclusters in DS, DFUNH, and DFUH. Subclusters 1, 5, and 9 showed higher proportions in DFUNH, while their proportions decreased in DFUH (Fig 10B and 10C). On the other hand, subclusters 4 and 6 had lower proportions in DFUNH but showed a rebound in proportions in DFUH (Fig 10B and 10C). Since subclusters 4 and 6 mainly originated from the DFUH group (Fig 10C), we defined them as healing-related fibroblast subtypes. Similarly, we defined subclusters 1, 5, and 9 as rescue-down subtypes and subclusters 4 and 6 as rescue-up subtypes.

**Fig 12:**
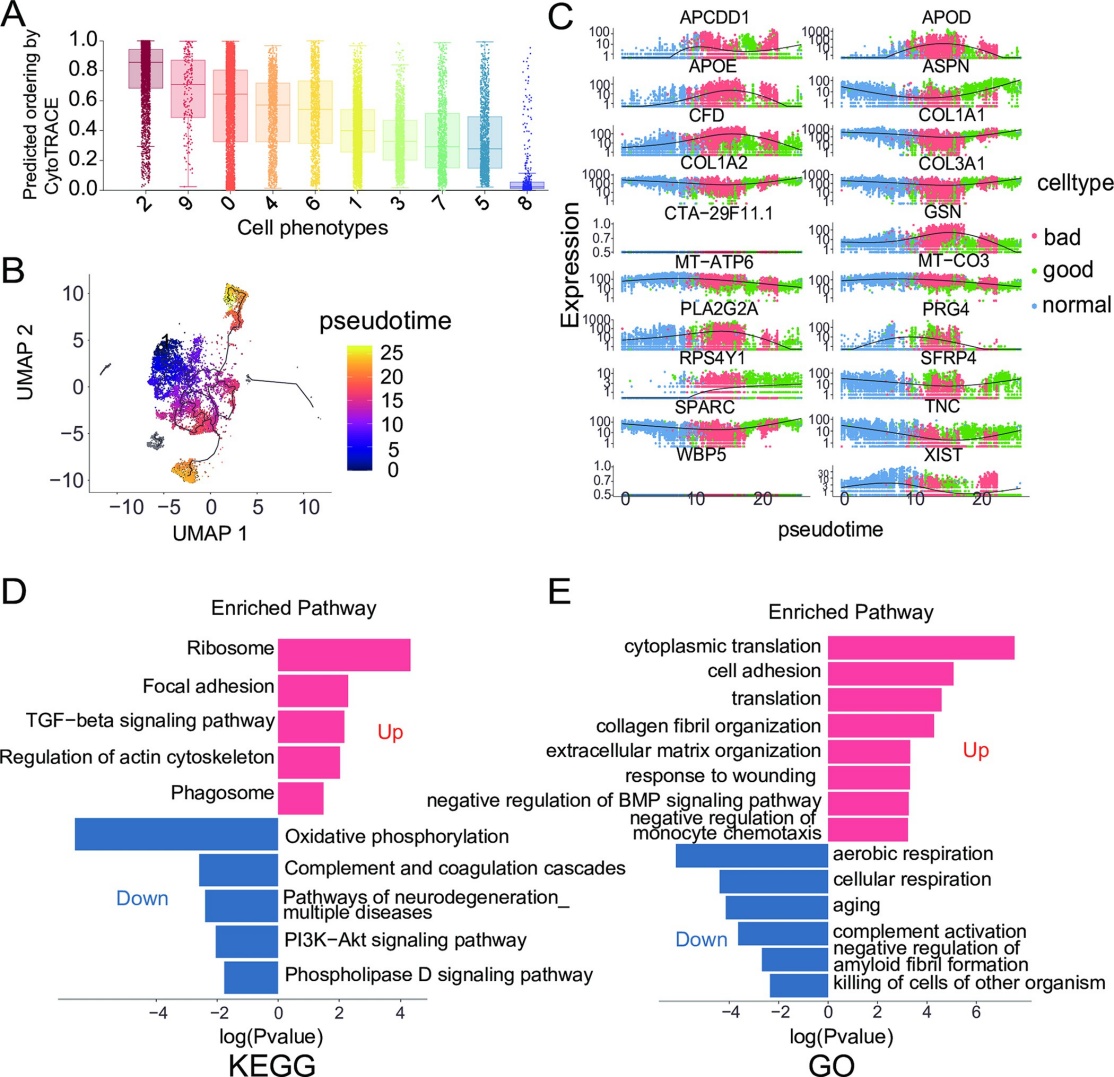
Pseudotemporal analysis of fibroblast subpopulations and analysis of biological differences among subpopulations. (A) Cytotrace determined the differentiation order or sequential appearance of subpopulations. Cytotrace scores from high to low correspond to the order of cell differentiation or sequential appearance. (B) Monocle3 inferred trajectories of fibroblast clusters. From deep purple to light yellow represents a putative temporal trajectory of fibroblast differentiation. (C) Genes significantly changed during the transition from a normal phenotype to a bad phenotype and then to a good phenotype. (D-E) KEGG and GO enrichment analysis of differentially expressed genes between good and bad cells. Red represents enrichment entries of upregulated genes in good cells, and blue represents enrichment entries of downregulated genes in good cells. The x-axis represents the log of the p-value corresponding to each enrichment entry. Positive values indicate upregulation, and negative values indicate downregulation.

To investigate the differentiation sequence of fibroblast subtypes, we used Cytotrace to predict the starting subcluster and the order of differentiation. The results showed that Cluster 2 was located at the starting point of differentiation, followed by clusters 9, 0, 4, 6, 1, 3, 7, 5, and 8 (Fig 12A). Assuming that clusters 1, 5, and 9 have a negative impact on wound healing, while clusters 4 and 6 have a positive impact, we referred to clusters 1, 5, and 9 as "bad" fibroblasts and clusters 4 and 6 as "good" fibroblasts (Fig 10D).

To explore the differences in the differentiation fate from classical fibroblasts to "bad" or "good" subtypes, we performed pseudo-time analysis. Cluster 0 represented classical fibroblast types, and its proportion did not significantly change among the groups. Therefore, we considered Cluster 0 as "normal" cells and used it as the starting point for pseudo-time analysis, while "bad" and "good" cells represented the two different differentiation fates (Fig 12B). Pseudo-time analysis results revealed significant gene expression changes during the transition from "normal" to "bad" or "good" cell phenotypes. Genes such as APCDD1, APOD, APOE, CFD, GSN, MT-ATP6, MT-CO3, PLA2G2A, and PRG4 were upregulated in the transition from "normal" to "bad" phenotype but showed a decreasing trend in expression during the transition from "bad" to "good" phenotype (Fig 12C). These genes with altered expression may be involved in the phenotypic transition of different fibroblast subtypes, influencing wound healing. Similarly, ASPN, COL1A1, COL1A2, COL3A1, SFRP4, SPARC, and TNC showed decreased expression in the transition from "normal" to "bad" phenotype but increased expression in the transition from "bad" to "good" phenotype (Fig 12C). The results of the ROC curve analysis indicated that APOD, APOE, COL3A1, PRG4, and TUBA1B have the potential to serve as biomarkers for non-healing DFUs (S8 Fig).

ASPN promotes ECM signaling, adhesion, and migration. ASPN, an ECM protein, has been found to inhibit NF-κB activity induced by TLR2 and TLR4 and the expression of pro-inflammatory cytokines in macrophages [75]. TLR4-mediated inflammation drives the synergistic effect of hypoxia and hyperglycemia on impaired diabetic wound healing [76]. Therefore, excessive expression of ASPN may be an important determinant of DFUs healing. In a recent study, a distinct cluster of fibroblasts enriched in ASPN and POSTN was described as interstitial cells and demonstrated a more dermal localization [77]. The roles of COL1A1, COL1A2, and TNC in promoting wound healing have been described previously and are not further elaborated here. Studies have shown that wound healing is significantly delayed in SPARC-deficient mice, indicating the positive role of SPARC in wound healing [78].

We further investigated the biological functional differences between "good" and "bad" cells. GO and Kyoto Encyclopedia of Genes and Genomes (KEGG) enrichment analyses were performed for differentially expressed genes between these two cell types. GO enrichment analysis revealed that upregulated genes in "good" cells, compared to "bad" cells, were involved in cell adhesion, cytoplasmic translation, collagen fibril organization, extracellular matrix organization, response to wounding, negative regulation of BMP signaling pathway, and negative regulation of monocyte chemotaxis. Downregulated genes were associated with cellular respiration, aging, complement activation, negative regulation of amyloid fibril formation, and killing of cells of other organisms (Fig 12E). Aging prolongs the inflammatory phase and increases the production of reactive oxygen species (ROS), leading to further impairment of chronic wound healing [79]. Increased levels of C3, C3a, and C3d have been observed in chronic wounds, burns, or traumatic wounds [80–84], indicating uncontrolled complement activation in these types of wounds. Additionally, studies on animals lacking complement components and patients receiving complement inhibitors have confirmed the importance of controlling the complement system in wound healing [85–90]. Activation of the complement system in chronic wounds leads to cell death and enhanced inflammation, resulting in further damage and impaired wound healing [91]. Attenuation of complement activation through specific inhibitors is considered an innovative wound care strategy. Furthermore, KEGG enrichment analysis revealed that upregulated genes were involved in focal adhesion, TGF-beta signaling pathway, regulation of actin cytoskeleton, and phagosome, while downregulated genes were associated with oxidative phosphorylation, complement and coagulation cascades, and the PI3K-AKT signaling pathway (Fig 12D). Transforming growth factor-beta (TGFβ) plays a crucial role in maintaining skin homeostasis and is important for reepithelialization, inflammation, angiogenesis, and granulation tissue formation during wound healing. Numerous studies have shown that exogenous application of recombinant TGFβ1 accelerates healing in animal models [92, 93], while exogenous inhibition of TGFβ signaling using antibodies targeting all TGFβ subtypes impairs epithelialization and granulation tissue formation [94].

## Discussion

Here, we reexamined the single-cell atlas of distinct healing fates in diabetic foot ulcers (DFUs). We observed changes in the cellular composition, gene expression, and intercellular communication associated with non-healing wounds, providing new insights into the mechanisms underlying DFUs non-healing. Furthermore, we conducted single-cell analysis of DFUs patients with healing ulcers and elucidated the mechanisms underlying the phenotypic variations between healing and non-healing DFUs. In summary, our findings at the molecular and cellular levels identified a series of features associated with non-healing that are reversed during the healing process. These features include compromised systemic antigen processing and presentation related to HLA, dysregulated inflammation, and inactivation of ECM-receptor signaling.

Drawing immune landscapes for healers and non-healers revealed a higher abundance of M1 macrophages in healers and a higher abundance of M2 macrophages in non-healers. These findings seem contradictory to previous research. Generally, M1 macrophages promote inflammatory responses, while M2 macrophages primarily inhibit inflammation and promote angiogenesis. In diabetic wounds, there is a persistent excessive inflammation, with M1 macrophages persisting in the wound bed and scarce M2 macrophages [95]. The favorable outcome of wound healing depends on the highly regulated balance of macrophage polarization states [96]. However, the presence of more M2 macrophages does not necessarily indicate better healing, as studies have shown delayed healing in diabetic mice treated with M2 macrophages [97, 98]. Interestingly, healers had lower negative regulation scores for local inflammatory responses in foot ulcer sites, while higher negative regulation scores for systemic inflammation in peripheral blood, suggesting the suppression of local inflammation activation and systemic inflammation in healers. These findings highlight the fundamental differences between systemic and local wound inflammation environments. Overall, our results provide further evidence supporting the notion that locally activated inflammatory response is essential to overcome chronic inflammation in DFUs and progress to the next stage of wound healing [99, 100]. Conversely, inhibiting inflammation processes at the systemic level seems beneficial for healing.

Abnormal production and remodeling of the extracellular matrix (ECM) lead to tissue dysfunction and delayed healing. Our findings revealed disrupted ECM-receptor signaling in DFUs non-healers, and targeting these disrupted signals may contribute to improving the healing of diabetic foot ulcers. Given the promising prospects of materials science in DFUs research, especially the achievements in novel wound materials based on ECM preparation, our results may provide valuable references for the design and preparation of improved ECM materials.

Analysis of fibroblast subpopulations revealed that dermal fibroblasts are a diverse and highly heterogeneous group that plays different functional roles in wound healing. Fibroblasts at inflamed sites, such as those within tertiary lymphoid structures, have been shown to possess immune cell characteristics [101], while a significant proportion of fibroblasts in granulation tissue in mouse wounds originate from the myeloid lineage [102]. Our data suggest that specific fibroblast subtypes are key factors in DFUs healing, and targeting them may be a therapeutic option. However, the factors contributing to the heterogeneity of dermal fibroblast subpopulations remain unclear, and further research is needed to elucidate the origin of wound fibroblast heterogeneity.

Future longitudinal studies involving DFUs samples collected at multiple time points during the wound healing process from the same patients may help establish a map of the diabetic wound healing timeline [103]. However, obtaining high-quality DFUs samples consistently poses technical challenges, and single-nucleus sequencing may be an alternative and complementary method to provide single-cell resolution without relying on highly feasible single-cell suspensions.

## Conclusion

Our analysis provides additional insights into the microenvironment of wound healing, identifying cell types and signaling pathways that may be crucial in promoting DFUs healing. It serves as an important complement to DFUs single-cell sequencing studies, offering a valuable contribution in understanding the mechanisms involved in DFUs healing.

## Supporting information

S1 Fig Data normalization and principal component analysis (PCA). (A, C) Fig A (GSE134431) and Fig C (GSE28914) show the data before normalization (left) and after normalization (right). (B, D) PCA plots. The PCA plots depict the distribution of samples based on gene expression profiles. Each point represents a sample, and the color of the points indicates the sample group. The distance between points reflects the similarity between samples. The first principal component (Dim1) and the second principal component (Dim2) are displayed on the X-axis and Y-axis, respectively. The plot clearly demonstrates the separation between sample groups based on gene expression profiles. (B) PCA plot of GSE134431. (D) PCA plot of GSE28914. DS: Diabetic Skin (no ulcer); DFUH: Diabetic Foot Ulcer (healed); DFUNH: Diabetic Foot Ulcer (non-healed). 0, 1, 3, and 7 represent days 0, 1, 3, and 7 of acute wound healing, respectively.
(TIF)

S2 Fig Violin plots showing UMI counts, gene counts, and mitochondrial UMI percentages of all quality-controlled cells from different clinical groups in blood (A) and skin (B). (TIF)

S3 Fig Identification of skin and peripheral blood cell types. (A, E) UMAP projections of clusters (colored markers) in the skin (A) and peripheral blood (E) at resolutions of 0.6 and 0.2. A total of 29 (skin) and 17 (peripheral blood) cell subgroups were identified in the skin and peripheral blood, respectively. (B, F) Expression distributions of cell marker genes in each cluster of the skin (B) and peripheral blood (F) at resolutions of 0.6 and 0.2. The size of the dots represents the percentage of cells expressing the marker gene in each cell group, while the color represents the average proportional expression level, with dark blue indicating low expression and yellow indicating high expression. (C, G) Dot plots showing the expression of cell type-specific marker genes used for annotating cell types. The size of the dots represents the percentage of cells expressing the marker gene in each cell group, while the color represents the average proportional expression level, with dark blue indicating low expression and yellow indicating high expression. Fifteen cell types were identified in the skin, and thirteen cell types were identified in the peripheral blood. (D, H) Heatmaps showing the top two highest expressed genes (in red) in each cell cluster. Red indicates high expression, while blue-purple indicates low expression.
(TIF)

S4 Fig Comparative analysis of cell types in different clinical groups. Group scatter plots represent the percentage of each cell type in clinical groups from peripheral blood and skin samples. The x-axis represents the clinical groups, and the y-axis represents the cell proportion. Each colored dot represents a sample. The solid gray dots represent the means, and the gray lines represent the upper and lower quartiles. The connecting lines between groups represent the corresponding P-values for intergroup comparisons. (A) The data represent the differences in cell proportions among the clinical groups of skin samples, including n = 8 patients with diabetes without foot ulcers, n = 9 patients with healed ulcers, and n = 5 patients with non-healing ulcers. (B) The data represent the differences in cell proportions among the clinical groups of peripheral blood samples, including n = 2 patients with diabetes without foot ulcers, n = 3 patients with healed ulcers, and n = 2 patients with non-healing ulcers. Statistical analysis was performed using t-tests. Cell types marked in red indicate statistically significant differences between groups (P<0.05). These differences may be confounded by the sample size of each clinical group and the variability in the number of single cells captured per sample. Due to the lack of distribution of granulocyte-monocyte progenitors in DS and DFUNH in peripheral blood, the scatter plot for this cell type is not displayed.
(TIF)

S5 Fig Identification of peripheral T cell subpopulations. (A) UMAP projection of peripheral blood T cell subgroups (different color annotations) when the resolution is set to 0.1. A total of 5 cell clusters were generated. (B) Expression distributions of cell marker genes for T cell subgroups in each cluster when the resolution is set to 0.1. The size of the dots represents the percentage of cells expressing the marker gene in each cell group, while the color represents the average proportional expression level, with dark blue indicating low expression and yellow indicating high expression. (C) Uniform Manifold Approximation and Projection (UMAP) embeddings generated from datasets containing 3368 cells. Cells are colored based on orthogonal-generated clusters and annotated manually according to their cell types (CD8+ T cells: CD8_T; CD4+ T cells: CD4_T). (D) Represents the average values and upper/lower quartiles of T cell subgroup cell proportions in peripheral blood for n = 2 diabetic patients without foot ulcers, n = 3 ulcer healing patients, and n = 2 non-healing ulcer patients. Statistical analysis was performed using a t-test.
(TIF)

S6 Fig Cell distribution of unhealing (A, D), healing (B, E), and rescue DEGs (C, F) in the skin (A, B, C) and peripheral blood (D, E, F). The gray outer circle represents the union of unhealing, healing, and rescue DEGs, while the different solid colored circles inside the circle represent each distinct cell type. Each cell type is connected to its corresponding DEGs by internal lines in the network. The bar chart on the right side of each cell-network diagram shows the number of DEGs contained in each cell type.
(PDF)

S7 Fig The gene expression scores of the GO terms "antigen processing and presentation" and "response to interferon beta" in the three clinical groups. (A) Gene expression scores of "antigen processing and presentation" in the single-cell datasets of the DS, DFUNH, and DFUH groups. *, P<0.05; ****, P<0.0001. (B) Gene expression scores of "response to interferon beta" in the single-cell datasets of the DS, DFUNH, and DFUH groups. ****, P<0.0001.
(PDF)

S8 Fig ROC curve for some genes in the GSE134431 dataset. In the GSE134431 dataset, the gene expression matrix of non-healing DFU and healing DFU samples was used to evaluate the AUC (Area Under the Curve) values of the ROC curves for the following genes: SFRP4, PLA2G2A, APCDD1, APOD, APOE, ASPN, CD74, CFD, COL1A1, COL1A2, COL3A1, COL6A1, COL6A2, FN1, GSN, HLA-B, HLA-C, HLA-DRA, MMP14, MMP2, PRG4, SPARC, THBS1, THBS2, TIMP1, TIMP2, TNC, TNXB, and TUBA1B. The AUC value reflects the accuracy of the prediction, with a higher value indicating a higher accuracy. The closer the curve is to the upper left corner (smaller X and larger Y), the higher the prediction accuracy. AUC values greater than 0.7 are considered to have good classification performance.
(PDF)

S9 Fig Ligand-receptor pairs of ECM-receptor signaling that are upregulated or downregulated during the Unhealing (DFUNH vs DS) and Healing (DFUH vs DFUNH) processes in the skin. (A) Cell origins of downregulated and upregulated ligand-receptor pairs during the Unhealing process. Blue represents cells from the DFUNH group, and red represents cells from the DS group. (B) Cell origins of downregulated and upregulated ligand-receptor pairs during the Healing process. Blue represents cells from the DFUH group, and red represents cells from the DFUNH group.
(PDF)

S10 Fig Expression patterns of TIMP1 (A), TIMP2 (B), MMP2 (C) and MMP14 (D) in various cell types within the skin tissue of the three clinical groups. *, P < 0.05; **, P < 0.01; P < 0.001; ****, P < 0.0001.
(PDF)

S1 Table Description of the public data set. DS, diabetic skin; DFUNH, diabetic foot ulcer (non-healed); DFUH, diabetic foot ulcer.
(XLS)

S2 Table Skin and peripheral blood cell marker. (XLSX)

S3 Table Rescue up ECM pathways (DFUNH/DS down, DFUH/DFUNH up). (XLSX)

S4 Table Rescue down ECM pathways (DFUNH/DS up, DFUH/DFUNH down). (XLSX)

## References

[1] Mottola C, Semedo-Lemsaddek T, Mendes JJ, Melo-Cristino J, Tavares L, Cavaco-Silva P, et al. Molecular typing, virulence traits and antimicrobial resistance of diabetic foot staphylococci. J Biomed Sci. 2016;23:33-. doi: 10.1186/s12929-016-0250-7 . 26952716 PMC4782296

[2] Janka-Zires M, Almeda-Valdes P, Uribe-Wiechers AC, Juárez-Comboni SC, López-Gutiérrez J, Escobar-Jiménez JJ, et al. Topical Administration of Pirfenidone Increases Healing of Chronic Diabetic Foot Ulcers: A Randomized Crossover Study. J Diabetes Res. 2016;2016:7340641. Epub 2016/08/02. doi: 10.1155/2016/7340641 ; PubMed Central PMCID: PMC4958428. 27478849 PMC4958428

[3] Dinh T, Tecilazich F, Kafanas A, Doupis J, Gnardellis C, Leal E, et al. Mechanisms Involved in the Development and Healing of Diabetic Foot Ulceration. Diabetes. 2012;61(11):2937–47. doi: 10.2337/db12-0227 22688339 PMC3478547

[4] Lerman OZ, Galiano RD, Armour M, Levine JP, Gurtner GC. Cellular dysfunction in the diabetic fibroblast: impairment in migration, vascular endothelial growth factor production, and response to hypoxia. Am J Pathol. 2003;162(1):303–12. Epub 2003/01/01. doi: 10.1016/S0002-9440(10)63821-7 ; PubMed Central PMCID: PMC1851127. 12507913 PMC1851127

[5] Hosseini Mansoub N. The role of keratinocyte function on the defected diabetic wound healing. Int J Burns Trauma. 2021;11(6):430–41. Epub 2022/02/04. ; PubMed Central PMCID: PMC8784740. 35111377 PMC8784740

[6] Geng K, Ma X, Jiang Z, Huang W, Gao C, Pu Y, et al. Innate Immunity in Diabetic Wound Healing: Focus on the Mastermind Hidden in Chronic Inflammatory. Front Pharmacol. 2021;12:653940. Epub 2021/05/11. doi: 10.3389/fphar.2021.653940 ; PubMed Central PMCID: PMC8097165. 33967796 PMC8097165

[7] Theocharidis G, Thomas BE, Sarkar D, Mumme HL, Pilcher WJR, Dwivedi B, et al. Single cell transcriptomic landscape of diabetic foot ulcers. Nat Commun. 2022;13(1):181. Epub 2022/01/12. doi: 10.1038/s41467-021-27801-8 ; PubMed Central PMCID: PMC8748704. 35013299 PMC8748704

[8] Sawaya AP, Stone RC, Brooks SR, Pastar I, Jozic I, Hasneen K, et al. Deregulated immune cell recruitment orchestrated by FOXM1 impairs human diabetic wound healing. Nat Commun. 2020;11(1):4678. Epub 2020/09/18. doi: 10.1038/s41467-020-18276-0 ; PubMed Central PMCID: PMC7495445. 32938916 PMC7495445

[9] Nuutila K, Siltanen A, Peura M, Bizik J, Kaartinen I, Kuokkanen H, et al. Human skin transcriptome during superficial cutaneous wound healing. Wound Repair Regen. 2012;20(6):830–9. Epub 2012/10/23. doi: 10.1111/j.1524-475X.2012.00831.x . 23082929

[10] Stuart T, Butler A, Hoffman P, Hafemeister C, Papalexi E, Mauck WM, 3rd, et al. Comprehensive Integration of Single-Cell Data. Cell. 2019;177(7):1888–902.e21. Epub 2019/06/11. doi: 10.1016/j.cell.2019.05.031 ; PubMed Central PMCID: PMC6687398. 31178118 PMC6687398

[11] Hafemeister C, Satija R. Normalization and variance stabilization of single-cell RNA-seq data using regularized negative binomial regression. Genome Biology. 2019;20(1):296. doi: 10.1186/s13059-019-1874-1 31870423 PMC6927181

[12] Zhang X, Lan Y, Xu J, Quan F, Zhao E, Deng C, et al. CellMarker: a manually curated resource of cell markers in human and mouse. Nucleic Acids Res. 2019;47(D1):D721–d8. Epub 2018/10/06. doi: 10.1093/nar/gky900 ; PubMed Central PMCID: PMC6323899. 30289549 PMC6323899

[13] Solé-Boldo L, Raddatz G, Schütz S, Mallm J-P, Rippe K, Lonsdorf AS, et al. Single-cell transcriptomes of the human skin reveal age-related loss of fibroblast priming. Communications Biology. 2020;3(1):188. doi: 10.1038/s42003-020-0922-4 32327715 PMC7181753

[14] Shannon P, Markiel A, Ozier O, Baliga NS, Wang JT, Ramage D, et al. Cytoscape: a software environment for integrated models of biomolecular interaction networks. Genome Res. 2003;13(11):2498–504. Epub 2003/11/05. doi: 10.1101/gr.1239303 ; PubMed Central PMCID: PMC403769. 14597658 PMC403769

[15] Ernst J, Bar-Joseph Z. STEM: a tool for the analysis of short time series gene expression data. BMC Bioinformatics. 2006;7:191. Epub 2006/04/07. doi: 10.1186/1471-2105-7-191 ; PubMed Central PMCID: PMC1456994. 16597342 PMC1456994

[16] Jin S, Guerrero-Juarez CF, Zhang L, Chang I, Ramos R, Kuan C-H, et al. Inference and analysis of cell-cell communication using CellChat. Nature Communications. 2021;12(1):1088. doi: 10.1038/s41467-021-21246-9 33597522 PMC7889871

[17] Trapnell C, Cacchiarelli D, Grimsby J, Pokharel P, Li S, Morse M, et al. The dynamics and regulators of cell fate decisions are revealed by pseudotemporal ordering of single cells. Nat Biotechnol. 2014;32(4):381–6. Epub 2014/03/25. doi: 10.1038/nbt.2859 ; PubMed Central PMCID: PMC4122333. 24658644 PMC4122333

[18] Gulati GS, Sikandar SS, Wesche DJ, Manjunath A, Bharadwaj A, Berger MJ, et al. Single-cell transcriptional diversity is a hallmark of developmental potential. Science. 2020;367(6476):405–11. Epub 2020/01/25. doi: 10.1126/science.aax0249 ; PubMed Central PMCID: PMC7694873. 31974247 PMC7694873

[19] Robin X, Turck N, Hainard A, Tiberti N, Lisacek F, Sanchez JC, et al. pROC: an open-source package for R and S+ to analyze and compare ROC curves. BMC Bioinformatics. 2011;12:77. Epub 2011/03/19. doi: 10.1186/1471-2105-12-77 ; PubMed Central PMCID: PMC3068975. 21414208 PMC3068975

[20] Cialdai F, Risaliti C, Monici M. Role of fibroblasts in wound healing and tissue remodeling on Earth and in space. Front Bioeng Biotechnol. 2022;10:958381. Epub 2022/10/22. doi: 10.3389/fbioe.2022.958381 ; PubMed Central PMCID: PMC9578548. 36267456 PMC9578548

[21] Gregorio J, Meller S, Conrad C, Di Nardo A, Homey B, Lauerma A, et al. Plasmacytoid dendritic cells sense skin injury and promote wound healing through type I interferons. J Exp Med. 2010;207(13):2921–30. Epub 2010/12/01. doi: 10.1084/jem.20101102 ; PubMed Central PMCID: PMC3005239. 21115688 PMC3005239

[22] Lin S, Wang Q, Huang X, Feng J, Wang Y, Shao T, et al. Wounds under diabetic milieu: The role of immune cellar components and signaling pathways. BIOMEDICINE & PHARMACOTHERAPY. 2023;157:114052. doi: 10.1016/j.biopha.2022.114052 36462313

[23] Boyette LB, Macedo C, Hadi K, Elinoff BD, Walters JT, Ramaswami B, et al. Phenotype, function, and differentiation potential of human monocyte subsets. PLoS One. 2017;12(4):e0176460. Epub 2017/04/27. doi: 10.1371/journal.pone.0176460 ; PubMed Central PMCID: PMC5406034. 28445506 PMC5406034

[24] Rajesh A, Stuart G, Real N, Tschirley A, Ahn J, Wise L, et al. Skin antigen-presenting cells and wound healing: New knowledge gained and challenges encountered using mouse depletion models. Immunology. 2021;163(1):98–104. Epub 2021/01/27. doi: 10.1111/imm.13311 ; PubMed Central PMCID: PMC8044336. 33496963 PMC8044336

[25] Massa C, Wang Y, Marr N, Seliger B. Interferons and Resistance Mechanisms in Tumors and Pathogen-Driven Diseases-Focus on the Major Histocompatibility Complex (MHC) Antigen Processing Pathway. Int J Mol Sci. 2023;24(7). Epub 2023/04/14. doi: 10.3390/ijms24076736 ; PubMed Central PMCID: PMC10095295. 37047709 PMC10095295

[26] Crouse J, Kalinke U, Oxenius A. Regulation of antiviral T cell responses by type I interferons. NATURE REVIEWS IMMUNOLOGY. 2015;15(4):231–42. doi: 10.1038/nri3806 25790790

[27] Wolf SJ, Audu CO, Joshi A, denDekker A, Melvin WJ, Davis FM, et al. IFN-κ is critical for normal wound repair and is decreased in diabetic wounds. JCI Insight. 2022;7(9). Epub 2022/04/01. doi: 10.1172/jci.insight.152765 ; PubMed Central PMCID: PMC9090246. 35358091 PMC9090246

[28] Kanno E, Tanno H, Masaki A, Sasaki A, Sato N, Goto M, et al. Defect of Interferon γ Leads to Impaired Wound Healing through Prolonged Neutrophilic Inflammatory Response and Enhanced MMP-2 Activation. Int J Mol Sci. 2019;20(22). Epub 2019/11/16. doi: 10.3390/ijms20225657 ; PubMed Central PMCID: PMC6888635. 31726690 PMC6888635

[29] Touraine JL, Bétuel H, Pouteil-Noble C, Royo C. HLA class II antigens: structure, function, and expression in immunodeficiencies, autoimmune diseases, and allograft rejection. Adv Nephrol Necker Hosp. 1989;18:325–34. Epub 1989/01/01. . 2493721

[30] Khutsishvili KR, Magalashvili RD, Demetrashvili ZM, Gopodze LN. [HLA antigens and the process of operative wound healing]. Georgian Med News. 2006;(136):7–10. Epub 2006/08/15. . 16905834

[31] Qing C. The molecular biology in wound healing & non-healing wound. Chinese Journal of Traumatology. 2017;20(4):189–93. 10.1016/j.cjtee.2017.06.001. 28712679 PMC5555286

[32] Kim S-H, Turnbull J, Guimond S. Extracellular matrix and cell signalling: the dynamic cooperation of integrin, proteoglycan and growth factor receptor. Journal of Endocrinology. 2011;209(2):139–51. doi: 10.1530/JOE-10-0377 21307119

[33] Sutherland TE, Dyer DP, Allen JE. The extracellular matrix and the immune system: A mutually dependent relationship. Science. 2023;379(6633):eabp8964. doi: 10.1126/science.abp8964 36795835

[34] Huang Y, Kyriakides TR. The role of extracellular matrix in the pathophysiology of diabetic wounds. Matrix Biol Plus. 2020;6–7:100037. Epub 2021/02/06. doi: 10.1016/j.mbplus.2020.100037 ; PubMed Central PMCID: PMC7852307. 33543031 PMC7852307

[35] Hwang SJ, Ha GH, Seo WY, Kim CK, Kim K, Lee SB. Human collagen alpha-2 type I stimulates collagen synthesis, wound healing, and elastin production in normal human dermal fibroblasts (HDFs). BMB Rep. 2020;53(10):539–44. Epub 2020/08/28. doi: 10.5483/BMBRep.2020.53.10.120 ; PubMed Central PMCID: PMC7607150. 32843132 PMC7607150

[36] Ricard-Blum S. The collagen family. Cold Spring Harb Perspect Biol. 2011;3(1):a004978. Epub 2011/03/23. doi: 10.1101/cshperspect.a004978 ; PubMed Central PMCID: PMC3003457. 21421911 PMC3003457

[37] Ricard-Blum S, Ballut L. Matricryptins derived from collagens and proteoglycans. Front Biosci (Landmark Ed). 2011;16(2):674–97. Epub 2011/01/05. doi: 10.2741/3712 . 21196195

[38] Kisling A, Lust RM, Katwa LC. What is the role of peptide fragments of collagen I and IV in health and disease? Life Sci. 2019;228:30–4. Epub 2019/04/21. doi: 10.1016/j.lfs.2019.04.042 . 31004660

[39] Theocharidis G, Drymoussi Z, Kao AP, Barber AH, Lee DA, Braun KM, et al. Type VI Collagen Regulates Dermal Matrix Assembly and Fibroblast Motility. JOURNAL OF INVESTIGATIVE DERMATOLOGY. 2016;136(1):74–83. doi: 10.1038/JID.2015.352 26763426

[40] Sun S, Karsdal MA. Chapter 6—Type VI Collagen. In: Karsdal MA, editor. Biochemistry of Collagens, Laminins and Elastin: Academic Press; 2016. p. 49–55.

[41] Agah A, Kyriakides TR, Lawler J, Bornstein P. The lack of thrombospondin-1 (TSP1) dictates the course of wound healing in double-TSP1/TSP2-null mice. Am J Pathol. 2002;161(3):831–9. Epub 2002/09/06. doi: 10.1016/S0002-9440(10)64243-5 ; PubMed Central PMCID: PMC1867266. 12213711 PMC1867266

[42] Crawford SE, Stellmach V, Murphy-Ullrich JE, Ribeiro SM, Lawler J, Hynes RO, et al. Thrombospondin-1 is a major activator of TGF-beta1 in vivo. Cell. 1998;93(7):1159–70. Epub 1998/07/10. doi: 10.1016/s0092-8674(00)81460-9 . 9657149

[43] Kyriakides TR, Tam JW, Bornstein P. Accelerated wound healing in mice with a disruption of the thrombospondin 2 gene. J Invest Dermatol. 1999;113(5):782–7. Epub 1999/11/26. doi: 10.1046/j.1523-1747.1999.00755.x . 10571734

[44] Maclauchlan S, Skokos EA, Agah A, Zeng J, Tian W, Davidson JM, et al. Enhanced angiogenesis and reduced contraction in thrombospondin-2-null wounds is associated with increased levels of matrix metalloproteinases-2 and -9, and soluble VEGF. J Histochem Cytochem. 2009;57(4):301–13. Epub 2008/11/26. doi: 10.1369/jhc.2008.952689 ; PubMed Central PMCID: PMC2664984. 19029404 PMC2664984

[45] Ohashi T, Kiehart DP, Erickson HP. Dual labeling of the fibronectin matrix and actin cytoskeleton with green fluorescent protein variants. Journal of Cell Science. 2002;115(6):1221–9. doi: 10.1242/jcs.115.6.1221 11884521

[46] George EL, Georges-Labouesse EN, Patel-King RS, Rayburn H, Hynes RO. Defects in mesoderm, neural tube and vascular development in mouse embryos lacking fibronectin. Development. 1993;119(4):1079–91. doi: 10.1242/dev.119.4.1079 8306876

[47] Natal C, Osés-Prieto JA, Pelacho B, Iraburu MJ, López-Zabalza MJ. Regulation of apoptosis by peptides of fibronectin in human monocytes. Apoptosis. 2006;11(2):209–19. doi: 10.1007/s10495-006-3761-y 16502259

[48] To WS, Midwood KS. Plasma and cellular fibronectin: distinct and independent functions during tissue repair. Fibrogenesis & Tissue Repair. 2011;4(1):21. doi: 10.1186/1755-1536-4-21 21923916 PMC3182887

[49] Hynes RO. The Extracellular Matrix: Not Just Pretty Fibrils. Science. 2009;326(5957):1216–9. doi: 10.1126/science.1176009 19965464 PMC3536535

[50] Vogel V. Unraveling the Mechanobiology of Extracellular Matrix. Annual Review of Physiology. 2018;80(1):353–87. doi: 10.1146/annurev-physiol-021317-121312 29433414

[51] Smigiel KS, Parks WC. Macrophages, Wound Healing, and Fibrosis: Recent Insights. Curr Rheumatol Rep. 2018;20(4):17. Epub 2018/03/20. doi: 10.1007/s11926-018-0725-5 . 29550962

[52] Armstrong DG, Jude EB. The role of matrix metalloproteinases in wound healing. J Am Podiatr Med Assoc. 2002;92(1):12–8. Epub 2002/01/18. doi: 10.7547/87507315-92-1-12 . 11796794

[53] Falanga V. The chronic wound: impaired healing and solutions in the context of wound bed preparation. Blood Cells Mol Dis. 2004;32(1):88–94. Epub 2004/02/06. doi: 10.1016/j.bcmd.2003.09.020 . 14757419

[54] Martins VL, Caley M, O’Toole EA. Matrix metalloproteinases and epidermal wound repair. Cell Tissue Res. 2013;351(2):255–68. Epub 2012/04/25. doi: 10.1007/s00441-012-1410-z . 22526628

[55] Menke MN, Menke NB, Boardman CH, Diegelmann RF. Biologic therapeutics and molecular profiling to optimize wound healing. Gynecol Oncol. 2008;111(2 Suppl):S87–91. Epub 2008/10/03. doi: 10.1016/j.ygyno.2008.07.052 ; PubMed Central PMCID: PMC2592097. 18829090 PMC2592097

[56] Heljasvaara R, Nyberg P, Luostarinen J, Parikka M, Heikkilä P, Rehn M, et al. Generation of biologically active endostatin fragments from human collagen XVIII by distinct matrix metalloproteases. Exp Cell Res. 2005;307(2):292–304. Epub 2005/06/14. doi: 10.1016/j.yexcr.2005.03.021 . 15950618

[57] Kato T, Kure T, Chang JH, Gabison EE, Itoh T, Itohara S, et al. Diminished corneal angiogenesis in gelatinase A-deficient mice. FEBS Lett. 2001;508(2):187–90. Epub 2001/11/24. doi: 10.1016/s0014-5793(01)02897-6 . 11718713

[58] auf dem Keller U, Sabino F. Matrix metalloproteinases in impaired wound healing. MNM.1. doi: 10.2147/mnm.s68420

[59] Strongin AY, Collier I, Bannikov G, Marmer BL, Grant GA, Goldberg GI. Mechanism of cell surface activation of 72-kDa type IV collagenase. Isolation of the activated form of the membrane metalloprotease. The Journal of biological chemistry. 1995;270(10):5331–8. Epub 1995/03/10. doi: 10.1074/jbc.270.10.5331 . 7890645

[60] Holmbeck K, Bianco P, Caterina J, Yamada S, Kromer M, Kuznetsov SA, et al. MT1-MMP-deficient mice develop dwarfism, osteopenia, arthritis, and connective tissue disease due to inadequate collagen turnover. Cell. 1999;99(1):81–92. Epub 1999/10/16. doi: 10.1016/s0092-8674(00)80064-1 . 10520996

[61] Zhou Z, Apte SS, Soininen R, Cao R, Baaklini GY, Rauser RW, et al. Impaired endochondral ossification and angiogenesis in mice deficient in membrane-type matrix metalloproteinase I. Proc Natl Acad Sci U S A. 2000;97(8):4052–7. Epub 2000/03/29. doi: 10.1073/pnas.060037197 ; PubMed Central PMCID: PMC18145. 10737763 PMC18145

[62] Atkinson JJ, Toennies HM, Holmbeck K, Senior RM. Membrane type 1 matrix metalloproteinase is necessary for distal airway epithelial repair and keratinocyte growth factor receptor expression after acute injury. Am J Physiol Lung Cell Mol Physiol. 2007;293(3):L600–10. Epub 2007/06/15. doi: 10.1152/ajplung.00028.2007 . 17557804

[63] Koshikawa N, Giannelli G, Cirulli V, Miyazaki K, Quaranta V. Role of cell surface metalloprotease MT1-MMP in epithelial cell migration over laminin-5. J Cell Biol. 2000;148(3):615–24. Epub 2000/02/09. doi: 10.1083/jcb.148.3.615 ; PubMed Central PMCID: PMC2174802. 10662785 PMC2174802

[64] Kajita M, Itoh Y, Chiba T, Mori H, Okada A, Kinoh H, et al. Membrane-type 1 matrix metalloproteinase cleaves CD44 and promotes cell migration. J Cell Biol. 2001;153(5):893–904. Epub 2001/05/31. doi: 10.1083/jcb.153.5.893 ; PubMed Central PMCID: PMC2174329. 11381077 PMC2174329

[65] Endo K, Takino T, Miyamori H, Kinsen H, Yoshizaki T, Furukawa M, et al. Cleavage of syndecan-1 by membrane type matrix metalloproteinase-1 stimulates cell migration. J Biol Chem. 2003;278(42):40764–70. Epub 2003/08/09. doi: 10.1074/jbc.M306736200 . 12904296

[66] Mirastschijski U, Zhou Z, Rollman O, Tryggvason K, Agren MS. Wound healing in membrane-type-1 matrix metalloproteinase-deficient mice. J Invest Dermatol. 2004;123(3):600–2. Epub 2004/08/12. doi: 10.1111/j.0022-202X.2004.23230.x . 15304103

[67] Vaalamo M, Leivo T, Saarialho-Kere U. Differential expression of tissue inhibitors of metalloproteinases (TIMP-1, -2, -3, and -4) in normal and aberrant wound healing. Hum Pathol. 1999;30(7):795–802. Epub 1999/07/22. doi: 10.1016/s0046-8177(99)90140-5 . 10414498

[68] Vaalamo M, Weckroth M, Puolakkainen P, Kere J, Saarinen P, Lauharanta J, et al. Patterns of matrix metalloproteinase and TIMP-1 expression in chronic and normally healing human cutaneous wounds. Br J Dermatol. 1996;135(1):52–9. Epub 1996/07/01. . 8776359

[69] Lao G, Ren M, Wang X, Zhang J, Huang Y, Liu D, et al. Human tissue inhibitor of metalloproteinases-1 improved wound healing in diabetes through its anti-apoptotic effect. Experimental Dermatology. 2019;28(5):528–35. doi: 10.1111/exd.13442 28887854

[70] Cui N, Hu M, Khalil RA. Biochemical and Biological Attributes of Matrix Metalloproteinases. Prog Mol Biol Transl Sci. 2017;147:1–73. Epub 2017/04/18. doi: 10.1016/bs.pmbts.2017.02.005 ; PubMed Central PMCID: PMC5430303. 28413025 PMC5430303

[71] Lobmann R, Ambrosch A, Schultz G, Waldmann K, Schiweck S, Lehnert H. Expression of matrix-metalloproteinases and their inhibitors in the wounds of diabetic and non-diabetic patients. Diabetologia. 2002;45(7):1011–6. Epub 2002/07/24. doi: 10.1007/s00125-002-0868-8 . 12136400

[72] Terasaki K, Kanzaki T, Aoki T, Iwata K, Saiki I. Effects of recombinant human tissue inhibitor of metalloproteinases-2 (rh-TIMP-2) on migration of epidermal keratinocytes in vitro and wound healing in vivo. J Dermatol. 2003;30(3):165–72. Epub 2003/04/15. doi: 10.1111/j.1346-8138.2003.tb00367.x . 12692351

[73] Gulino GR, Magnetto C, Khadjavi A, Panariti A, Rivolta I, Soster M, et al. Oxygen-Loaded Nanodroplets Effectively Abrogate Hypoxia Dysregulating Effects on Secretion of MMP-9 and TIMP-1 by Human Monocytes. Mediators Inflamm. 2015;2015:964838. Epub 2015/04/17. doi: 10.1155/2015/964838 ; PubMed Central PMCID: PMC4386605. 25878404 PMC4386605

[74] Khadjavi A, Magnetto C, Panariti A, Argenziano M, Gulino GR, Rivolta I, et al. Chitosan-shelled oxygen-loaded nanodroplets abrogate hypoxia dysregulation of human keratinocyte gelatinases and inhibitors: New insights for chronic wound healing. Toxicol Appl Pharmacol. 2015;286(3):198–206. Epub 2015/05/06. doi: 10.1016/j.taap.2015.04.015 . 25937238

[75] Yamaba S, Yamada S, Kajikawa T, Awata T, Sakashita H, Tsushima K, et al. PLAP-1/Asporin Regulates TLR2- and TLR4-induced Inflammatory Responses. J Dent Res. 2015;94(12):1706–14. Epub 2015/09/25. doi: 10.1177/0022034515606859 . 26399972

[76] Portou MJ, Yu R, Baker D, Xu S, Abraham D, Tsui J. Hyperglycaemia and Ischaemia Impair Wound Healing via Toll-like Receptor 4 Pathway Activation in vitro and in an Experimental Murine Model. Eur J Vasc Endovasc Surg. 2020;59(1):117–27. Epub 2019/11/17. doi: 10.1016/j.ejvs.2019.06.018 . 31732468

[77] Solé-Boldo L, Raddatz G, Schütz S, Mallm JP, Rippe K, Lonsdorf AS, et al. Single-cell transcriptomes of the human skin reveal age-related loss of fibroblast priming. Commun Biol. 2020;3(1):188. Epub 2020/04/25. doi: 10.1038/s42003-020-0922-4 ; PubMed Central PMCID: PMC7181753 competing financial interests: F.L. received consultation fees from Beiersdorf AG. The other authors have no competing financial interests. 32327715 PMC7181753

[78] Basu A, Kligman LH, Samulewicz SJ, Howe CC. Impaired wound healing in mice deficient in a matricellular protein SPARC (osteonectin, BM-40). BMC Cell Biol. 2001;2:15. Epub 2001/09/05. doi: 10.1186/1471-2121-2-15 ; PubMed Central PMCID: PMC48139. 11532190 PMC48139

[79] Khalid KA, Nawi AFM, Zulkifli N, Barkat MA, Hadi H. Aging and Wound Healing of the Skin: A Review of Clinical and Pathophysiological Hallmarks. Life. 2022;12(12):2142. doi: 10.3390/life12122142 36556508 PMC9784880

[80] Balslev E, Thomsen HK, Danielsen L, Sheller J, Garred P. The terminal complement complex is generated in chronic leg ulcers in the absence of protectin (CD59). Apmis. 1999;107(7‐12):997–1004. doi: 10.1111/j.1699-0463.1999.tb01502.x 10598871

[81] Schmidtchen A. Degradation of antiproteinases, complement and fibronectin in chronic leg ulcers. ACTA DERMATOVENEREOLOGICA-STOCKHOLM-. 2000;80(3):179–84. doi: 10.1080/000155500750042925 10954207

[82] Jacobsen JN, Andersen AS, Sonnested MK, Laursen I, Jorgensen B, Krogfelt KA. Investigating the humoral immune response in chronic venous leg ulcer patients colonised with Pseudomonas aeruginosa. International wound journal. 2011;8(1):33–43. doi: 10.1111/j.1742-481X.2010.00741.x 21091636 PMC7950865

[83] Machens H-G, Pabst A, Dreyer M, Gliemroth J, Görg S, Bahlmann L, et al. C3a levels and occurrence of subdermal vascular thrombosis are age-related in deep second-degree burn wounds. Surgery. 2006;139(4):550–5. doi: 10.1016/j.surg.2005.09.001 16627066

[84] Wan K, Lewis W, Leung P, Chien P, Hung L. A longitudinal study of C3, C3d and factor Ba in burn patients in Hong Kong Chinese. Burns. 1998;24(3):241–4. doi: 10.1016/s0305-4179(98)00013-8 9677027

[85] Yeh C, Marsh H Jr, Carson G, Berman L, Concino M, Scesney S, et al. Recombinant soluble human complement receptor type 1 inhibits inflammation in the reversed passive arthus reaction in rats. Journal of immunology (Baltimore, Md: 1950). 1991;146(1):250–6. 1824590

[86] Wahl SM, Arend WP, Ross R. The effect of complement depletion on wound healing. The American Journal of Pathology. 1974;75(1):73. 4825618 PMC1910809

[87] Hew BE, Wehrhahn D, Fritzinger DC, Vogel C-W. Hybrid proteins of Cobra Venom Factor and cobra C3: tools to identify functionally important regions in Cobra Venom Factor. Toxicon. 2012;60(4):632–47. doi: 10.1016/j.toxicon.2012.05.004 22609532

[88] Henze U, Lennartz A, Hafemann B, Goldmann C, Kirkpatrick C, Klosterhalfen B. The influence of the C1-inhibitor BERINERT® and the protein-free haemodialysate ACTIHAEMYL20%® on the evolution of the depth of scald burns in a porcine model. Burns. 1997;23(6):473–7. doi: 10.1016/s0305-4179(97)00019-3 9429024

[89] Suber F, Carroll MC, Moore FD Jr. Innate response to self-antigen significantly exacerbates burn wound depth. Proceedings of the National Academy of Sciences. 2007;104(10):3973–7. doi: 10.1073/pnas.0609026104 17360462 PMC1820693

[90] Schmitt J, Roderfeld M, Sabrane K, Zhang P, Tian Y, Mertens JC, et al. Complement factor C5 deficiency significantly delays the progression of biliary fibrosis in bile duct-ligated mice. Biochemical and biophysical research communications. 2012;418(3):445–50. doi: 10.1016/j.bbrc.2012.01.036 22277671

[91] Cazander G, Jukema GN, Nibbering PH. Complement Activation and Inhibition in Wound Healing. Clinical and Developmental Immunology. 2012;2012:534291. doi: 10.1155/2012/534291 23346185 PMC3546472

[92] Puolakkainen PA, Reed MJ, Gombotz WR, Twardzik DR, Abrass IB, Sage HE. Acceleration of wound healing in aged rats by topical application of transforming growth factor-beta(1). Wound Repair Regen. 1995;3(3):330–9. Epub 1995/07/01. doi: 10.1046/j.1524-475X.1995.t01-1-30314.x . 17173560

[93] Mustoe TA, Pierce GF, Thomason A, Gramates P, Sporn MB, Deuel TF. Accelerated healing of incisional wounds in rats induced by transforming growth factor-beta. Science. 1987;237(4820):1333–6. Epub 1987/09/11. doi: 10.1126/science.2442813 . 2442813

[94] Lu L, Saulis AS, Liu WR, Roy NK, Chao JD, Ledbetter S, et al. The temporal effects of anti-TGF-beta1, 2, and 3 monoclonal antibody on wound healing and hypertrophic scar formation. J Am Coll Surg. 2005;201(3):391–7. Epub 2005/08/30. doi: 10.1016/j.jamcollsurg.2005.03.032 . 16125072

[95] Louiselle AE, Niemiec SM, Zgheib C, Liechty KW. Macrophage polarization and diabetic wound healing. Translational Research. 2021;236:109–16. doi: 10.1016/j.trsl.2021.05.006 34089902

[96] Spiller KL, Koh TJ. Macrophage-based therapeutic strategies in regenerative medicine. Adv Drug Deliv Rev. 2017;122:74–83. Epub 2017/05/21. doi: 10.1016/j.addr.2017.05.010 ; PubMed Central PMCID: PMC5690893. 28526591 PMC5690893

[97] Dreymueller D, Denecke B, Ludwig A, Jahnen-Dechent W. Embryonic stem cell-derived M2-like macrophages delay cutaneous wound healing. Wound Repair Regen. 2013;21(1):44–54. Epub 2012/11/07. doi: 10.1111/j.1524-475X.2012.00858.x . 23126541

[98] Jetten N, Roumans N, Gijbels MJ, Romano A, Post MJ, de Winther MPJ, et al. Wound Administration of M2-Polarized Macrophages Does Not Improve Murine Cutaneous Healing Responses. PLOS ONE. 2014;9(7):e102994. doi: 10.1371/journal.pone.0102994 25068282 PMC4113363

[99] Stone RC, Stojadinovic O, Rosa AM, Ramirez HA, Badiavas E, Blumenberg M, et al. A bioengineered living cell construct activates an acute wound healing response in venous leg ulcers. Sci Transl Med. 2017;9(371). Epub 2017/01/06. doi: 10.1126/scitranslmed.aaf8611 ; PubMed Central PMCID: PMC5472448. 28053158 PMC5472448

[100] Tellechea A, Bai S, Dangwal S, Theocharidis G, Nagai M, Koerner S, et al. Topical Application of a Mast Cell Stabilizer Improves Impaired Diabetic Wound Healing. J Invest Dermatol. 2020;140(4):901–11.e11. Epub 2019/10/01. doi: 10.1016/j.jid.2019.08.449 . 31568772

[101] Nayar S, Campos J, Smith CG, Iannizzotto V, Gardner DH, Mourcin F, et al. Immunofibroblasts are pivotal drivers of tertiary lymphoid structure formation and local pathology. Proc Natl Acad Sci U S A. 2019;116(27):13490–7. Epub 2019/06/20. doi: 10.1073/pnas.1905301116 ; PubMed Central PMCID: PMC6613169. 31213547 PMC6613169

[102] Sinha M, Sen CK, Singh K, Das A, Ghatak S, Rhea B, et al. Direct conversion of injury-site myeloid cells to fibroblast-like cells of granulation tissue. Nat Commun. 2018;9(1):936. Epub 2018/03/07. doi: 10.1038/s41467-018-03208-w ; PubMed Central PMCID: PMC5838200. 29507336 PMC5838200

[103] Nassiri S, Zakeri I, Weingarten MS, Spiller KL. Relative Expression of Proinflammatory and Antiinflammatory Genes Reveals Differences between Healing and Nonhealing Human Chronic Diabetic Foot Ulcers. J Invest Dermatol. 2015;135(6):1700–3. Epub 2015/02/04. doi: 10.1038/jid.2015.30 . 25647438

