# Supplementary figures and images for: Combined analysis of single-cell sequencing and bulk transcriptome sequencing reveals new mechanisms for non-healing diabetic foot ulcers
Source: PLoS One. 2024 Jul 1;19(7):e0306248. doi: 10.1371/journal.pone.0306248 (PMC11216623; doi:10.1371/journal.pone.0306248)

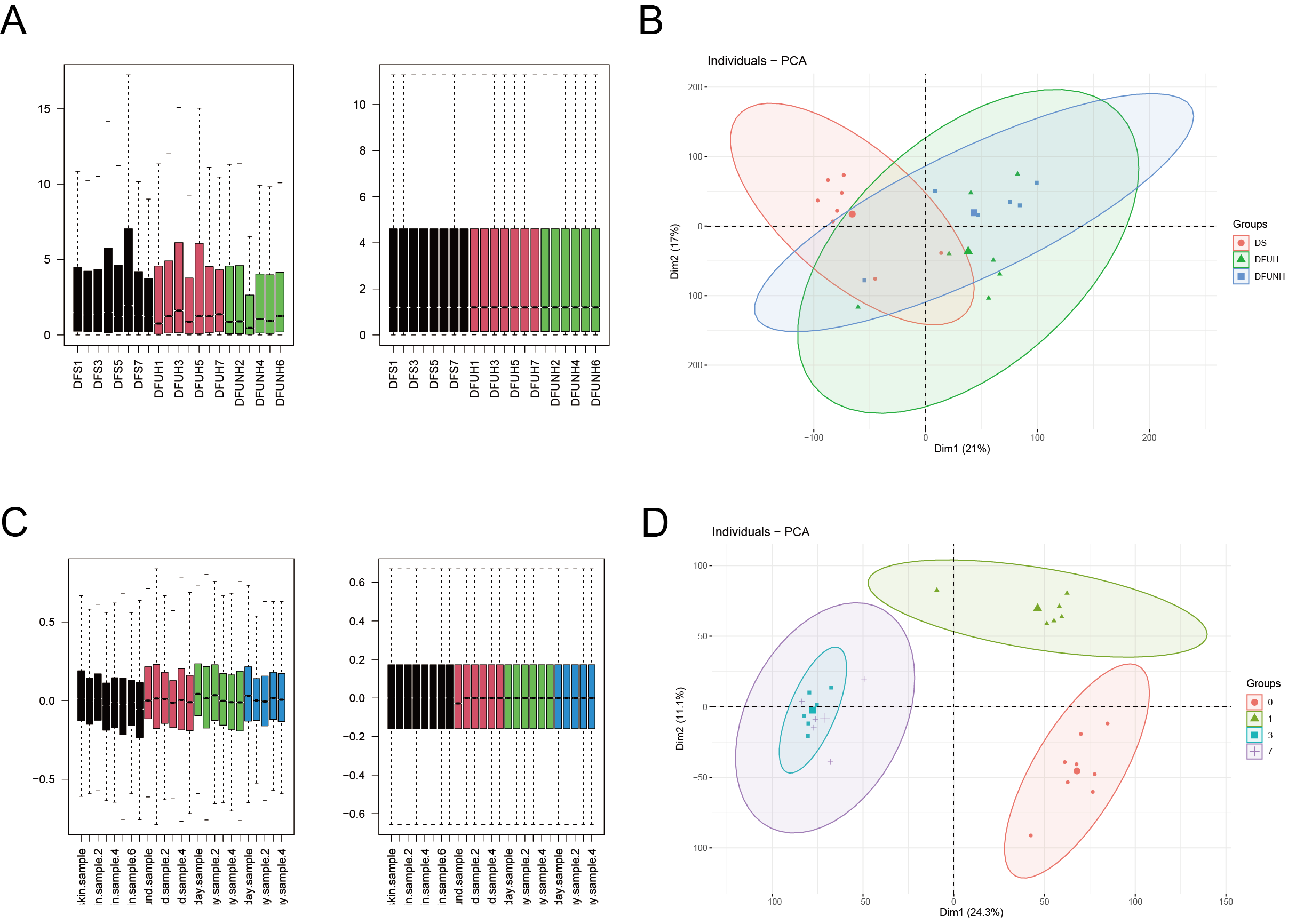

Supplement: S1 Fig — (A, C) Fig A (GSE134431) and Fig C (GSE28914) show the data before normalization (left) and after normalization (right). (B, D) PCA plots. The PCA plots depict the distribution of samples based on gene expression profiles. Each point represents a sample, and the color of the points indicates the sample group. The distance between points reflects the similarity between samples. The first principal component (Dim1) and the second principal component (Dim2) are displayed on the X-axis and Y-axis, respectively. The plot clearly demonstrates the separation between sample groups based on gene expression profiles. (B) PCA plot of GSE134431. (D) PCA plot of GSE28914. DS: Diabetic Skin (no ulcer); DFUH: Diabetic Foot Ulcer (healed); DFUNH: Diabetic Foot Ulcer (non-healed). 0, 1, 3, and 7 represent days 0, 1, 3, and 7 of acute wound healing, respectively. (TIF) [file pone.0306248.s001.tif]

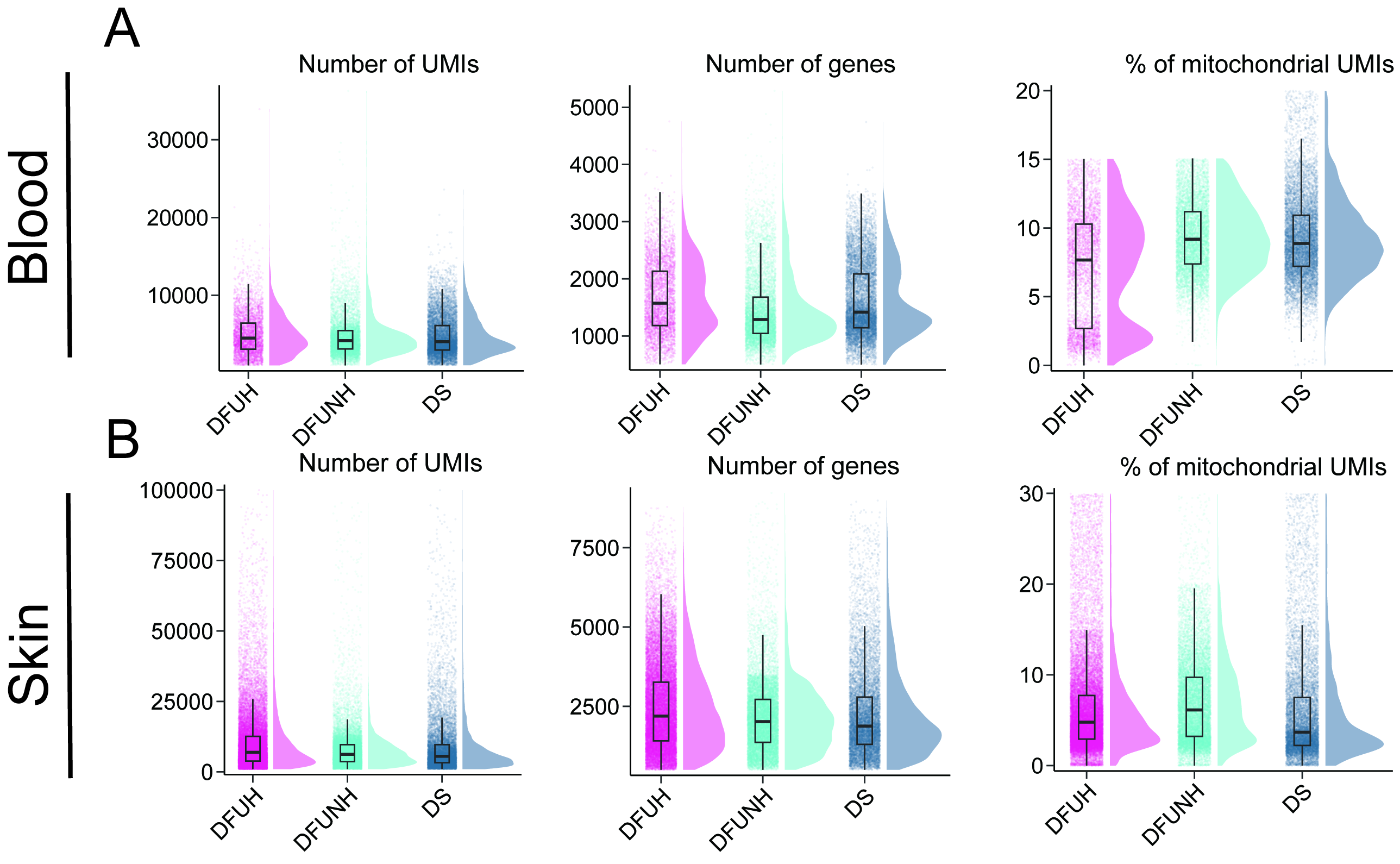

Supplement: S2 Fig — (TIF) [file pone.0306248.s002.tif]

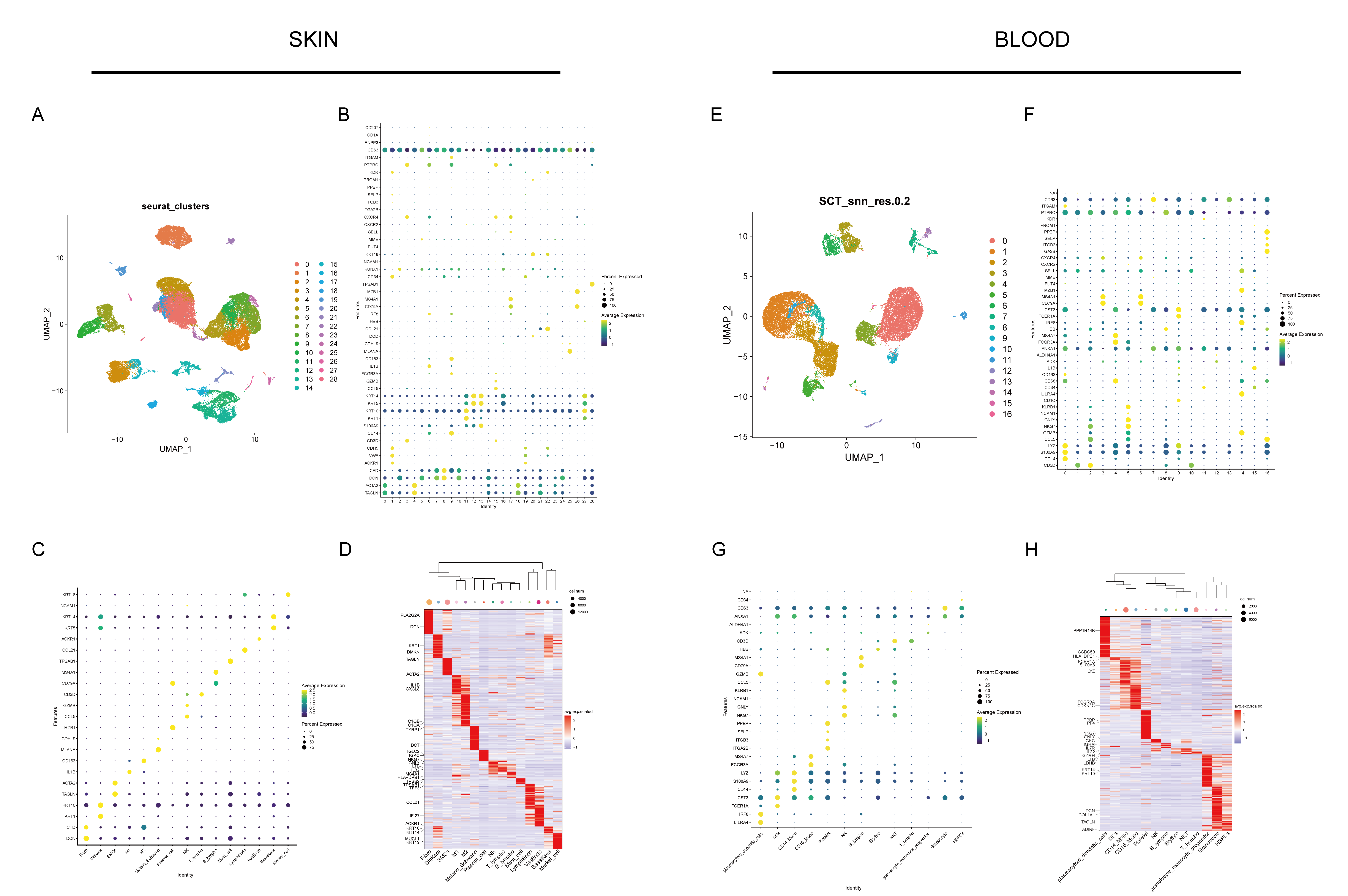

Supplement: S3 Fig — (A, E) UMAP projections of clusters (colored markers) in the skin (A) and peripheral blood (E) at resolutions of 0.6 and 0.2. A total of 29 (skin) and 17 (peripheral blood) cell subgroups were identified in the skin and peripheral blood, respectively. (B, F) Expression distributions of cell marker genes in each cluster of the skin (B) and peripheral blood (F) at resolutions of 0.6 and 0.2. The size of the dots represents the percentage of cells expressing the marker gene in each cell group, while the color represents the average proportional expression level, with dark blue indicating low expression and yellow indicating high expression. (C, G) Dot plots showing the expression of cell type-specific marker genes used for annotating cell types. The size of the dots represents the percentage of cells expressing the marker gene in each cell group, while the color represents the average proportional expression level, with dark blue indicating low expression and yellow indicating high expression. Fifteen cell types were identified in the skin, and thirteen cell types were identified in the peripheral blood. (D, H) Heatmaps showing the top two highest expressed genes (in red) in each cell cluster. Red indicates high expression, while blue-purple indicates low expression. (TIF) [file pone.0306248.s003.tif]

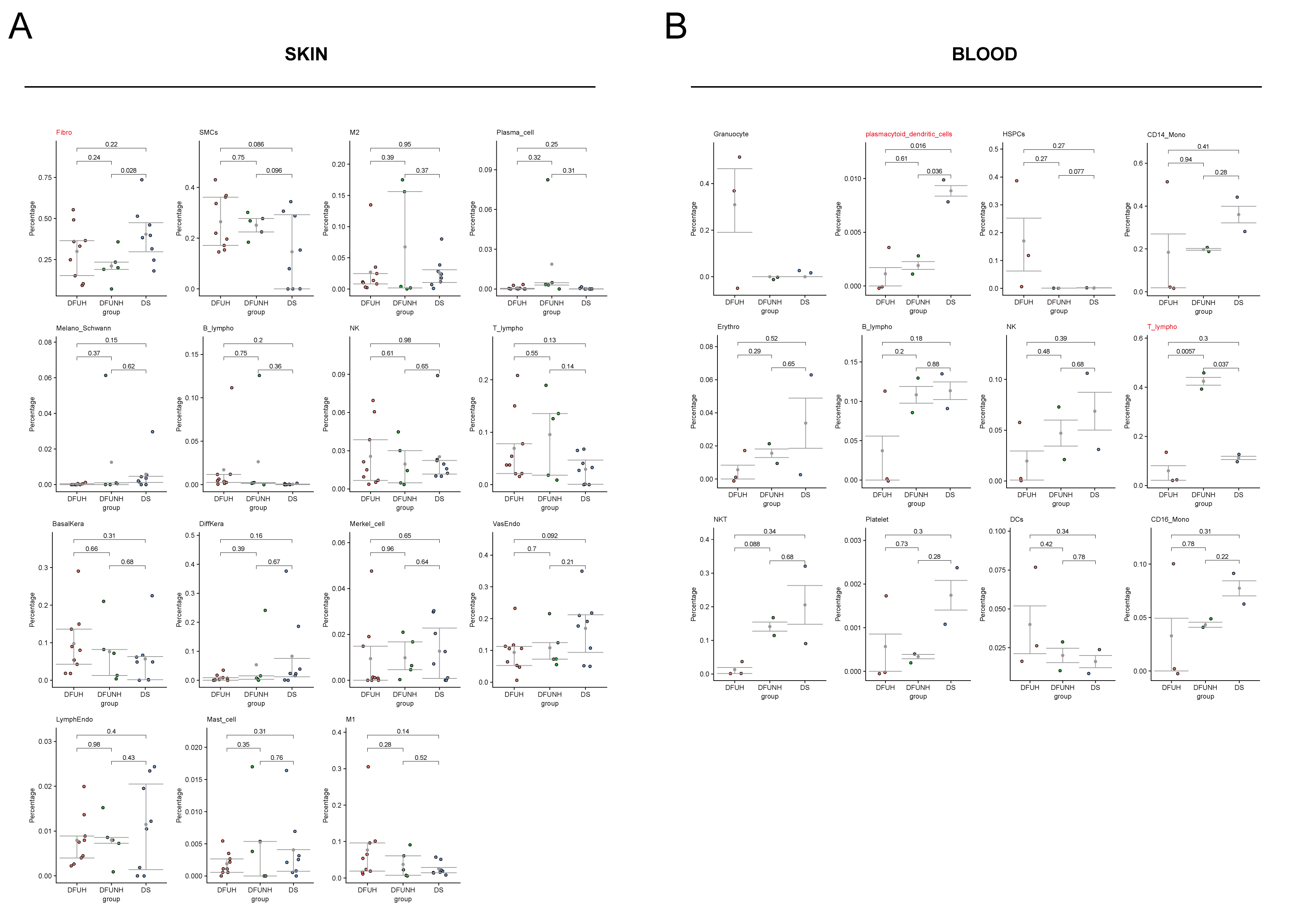

Supplement: S4 Fig — Group scatter plots represent the percentage of each cell type in clinical groups from peripheral blood and skin samples. The x-axis represents the clinical groups, and the y-axis represents the cell proportion. Each colored dot represents a sample. The solid gray dots represent the means, and the gray lines represent the upper and lower quartiles. The connecting lines between groups represent the corresponding P-values for intergroup comparisons. (A) The data represent the differences in cell proportions among the clinical groups of skin samples, including n = 8 patients with diabetes without foot ulcers, n = 9 patients with healed ulcers, and n = 5 patients with non-healing ulcers. (B) The data represent the differences in cell proportions among the clinical groups of peripheral blood samples, including n = 2 patients with diabetes without foot ulcers, n = 3 patients with healed ulcers, and n = 2 patients with non-healing ulcers. Statistical analysis was performed using t-tests. Cell types marked in red indicate statistically significant differences between groups (P<0.05). These differences may be confounded by the sample size of each clinical group and the variability in the number of single cells captured per sample. Due to the lack of distribution of granulocyte-monocyte progenitors in DS and DFUNH in peripheral blood, the scatter plot for this cell type is not displayed. (TIF) [file pone.0306248.s004.tif]

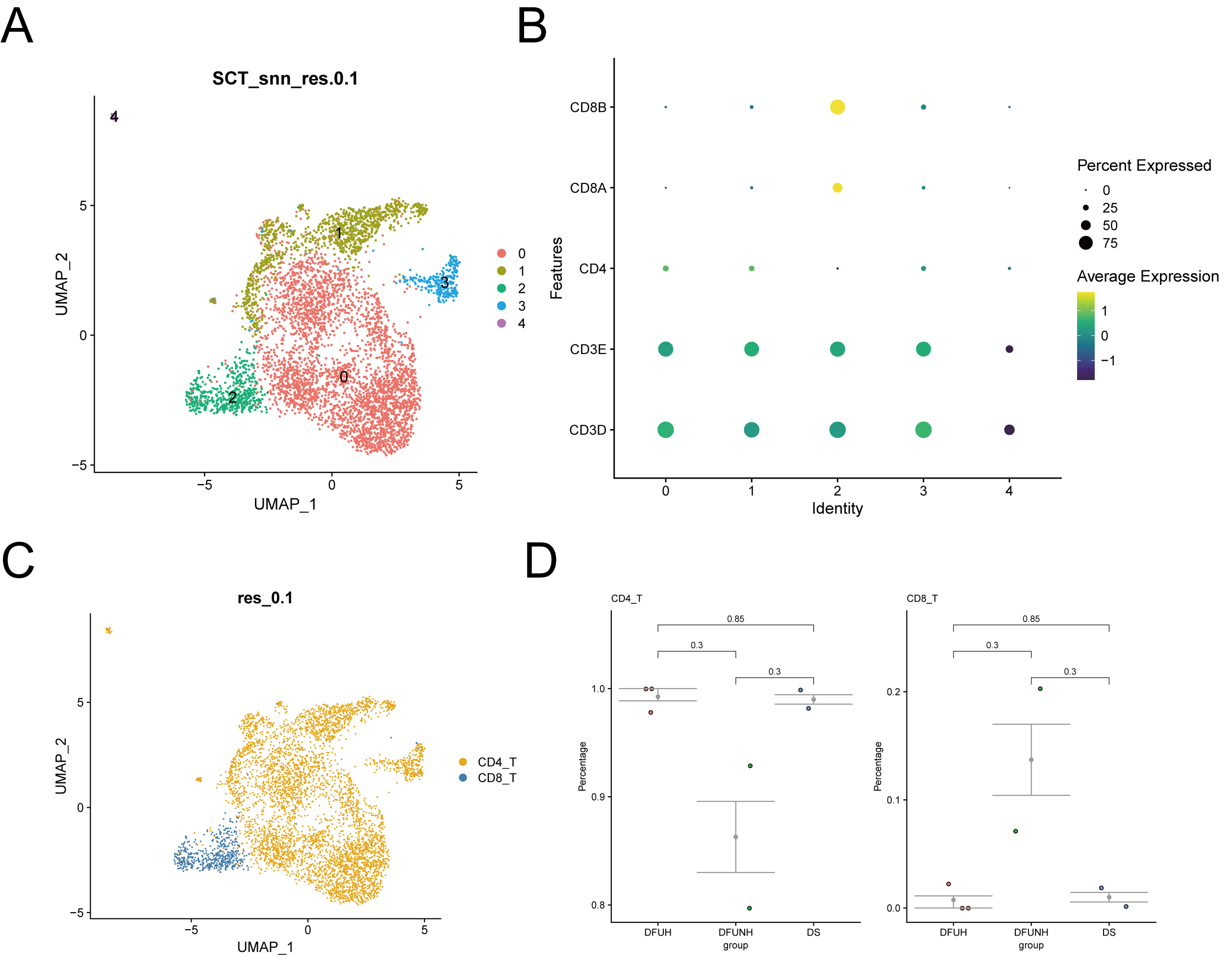

Supplement: S5 Fig — (A) UMAP projection of peripheral blood T cell subgroups (different color annotations) when the resolution is set to 0.1. A total of 5 cell clusters were generated. (B) Expression distributions of cell marker genes for T cell subgroups in each cluster when the resolution is set to 0.1. The size of the dots represents the percentage of cells expressing the marker gene in each cell group, while the color represents the average proportional expression level, with dark blue indicating low expression and yellow indicating high expression. (C) Uniform Manifold Approximation and Projection (UMAP) embeddings generated from datasets containing 3368 cells. Cells are colored based on orthogonal-generated clusters and annotated manually according to their cell types (CD8+ T cells: CD8_T; CD4+ T cells: CD4_T). (D) Represents the average values and upper/lower quartiles of T cell subgroup cell proportions in peripheral blood for n = 2 diabetic patients without foot ulcers, n = 3 ulcer healing patients, and n = 2 non-healing ulcer patients. Statistical analysis was performed using a t-test. (TIF) [file pone.0306248.s005.tif]

A

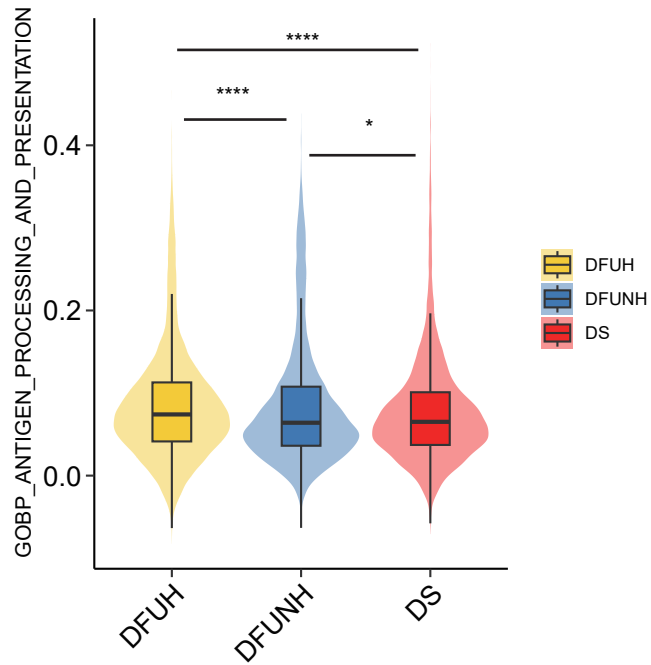

B

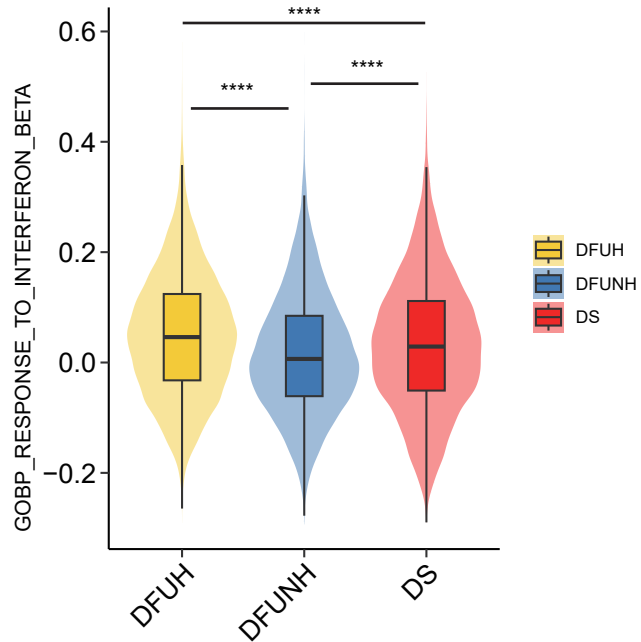

Supplement: S7 Fig — (A) Gene expression scores of "antigen processing and presentation" in the single-cell datasets of the DS, DFUNH, and DFUH groups. *, P<0.05; ****, P<0.0001. (B) Gene expression scores of "response to interferon beta" in the single-cell datasets of the DS, DFUNH, and DFUH groups. ****, P<0.0001. (PDF) [file pone.0306248.s007.pdf]

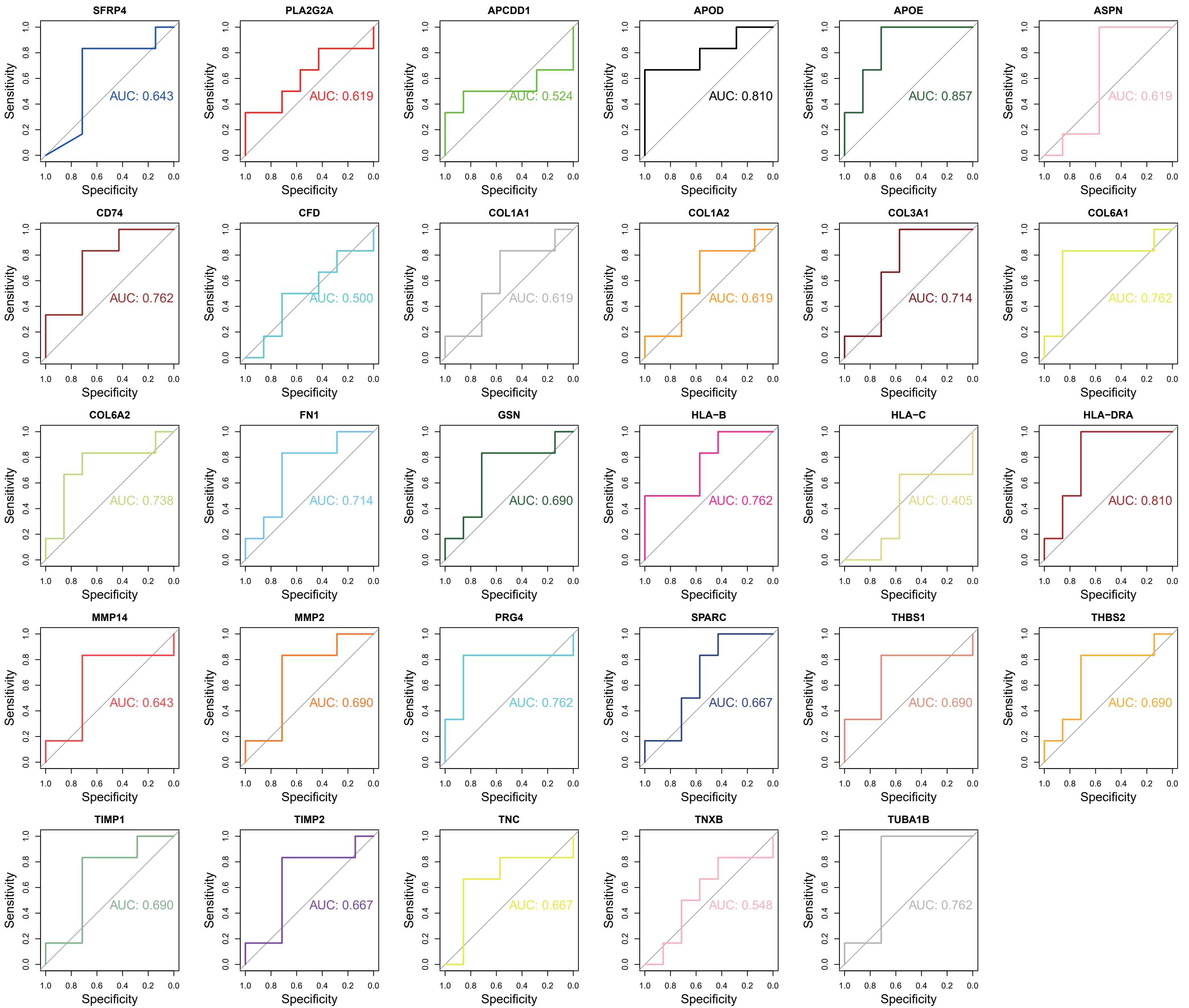

Supplement: S8 Fig — In the GSE134431 dataset, the gene expression matrix of non-healing DFU and healing DFU samples was used to evaluate the AUC (Area Under the Curve) values of the ROC curves for the following genes: SFRP4, PLA2G2A, APCDD1, APOD, APOE, ASPN, CD74, CFD, COL1A1, COL1A2, COL3A1, COL6A1, COL6A2, FN1, GSN, HLA-B, HLA-C, HLA-DRA, MMP14, MMP2, PRG4, SPARC, THBS1, THBS2, TIMP1, TIMP2, TNC, TNXB, and TUBA1B. The AUC value reflects the accuracy of the prediction, with a higher value indicating a higher accuracy. The closer the curve is to the upper left corner (smaller X and larger Y), the higher the prediction accuracy. AUC values greater than 0.7 are considered to have good classification performance. (PDF) [file pone.0306248.s008.pdf]

SKIN-ECM signaling

A

DFUNH vs DS

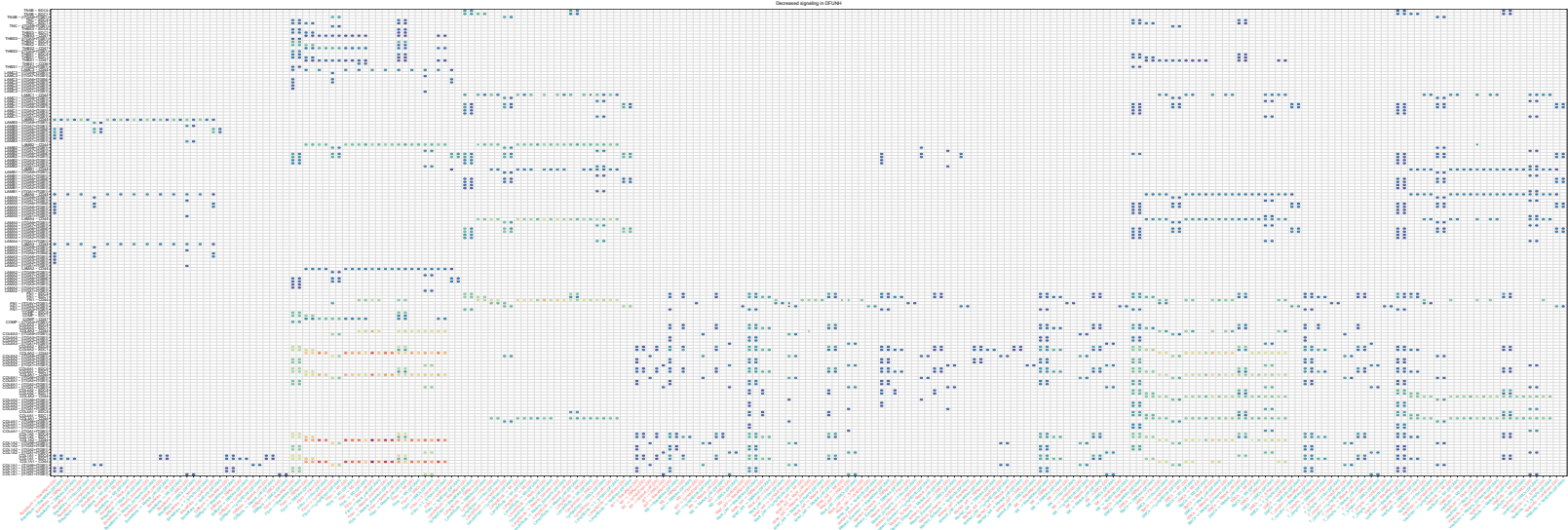

Down

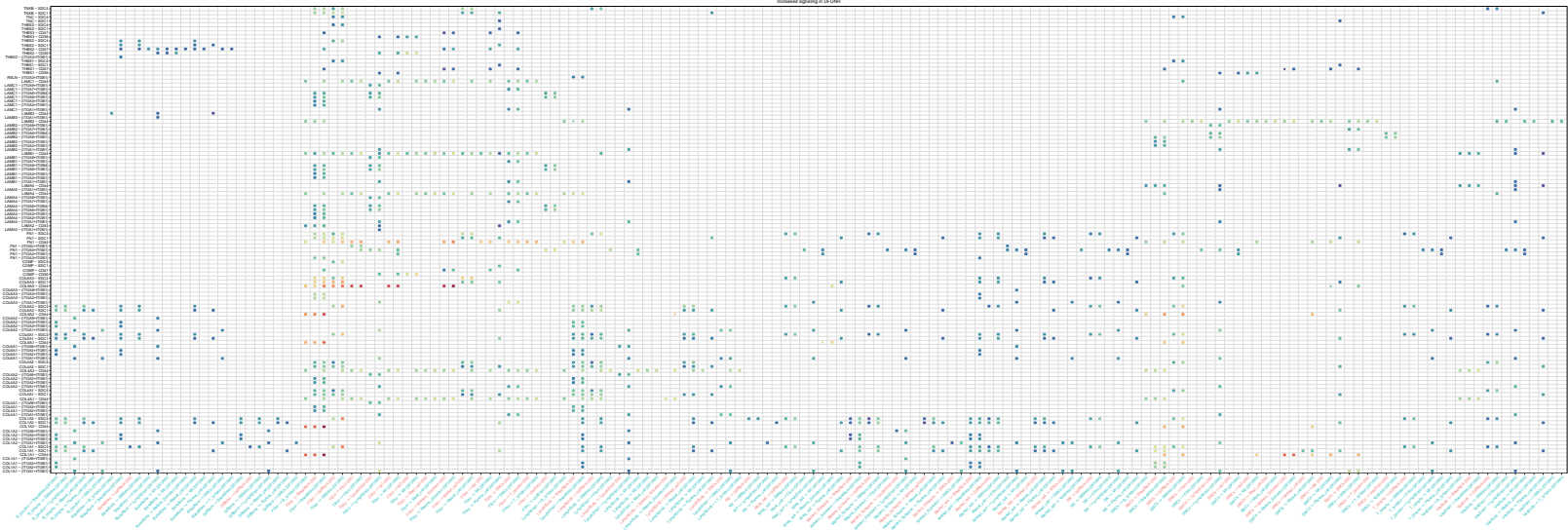

Up

B

DFUH vs DFUNH

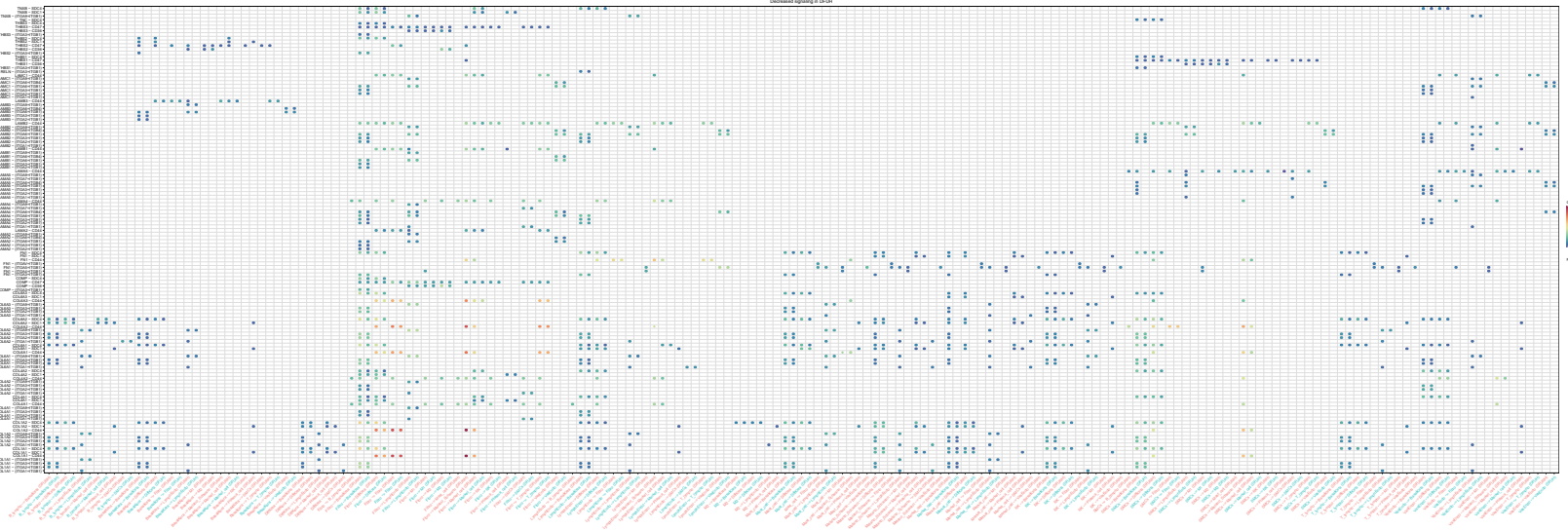

Down

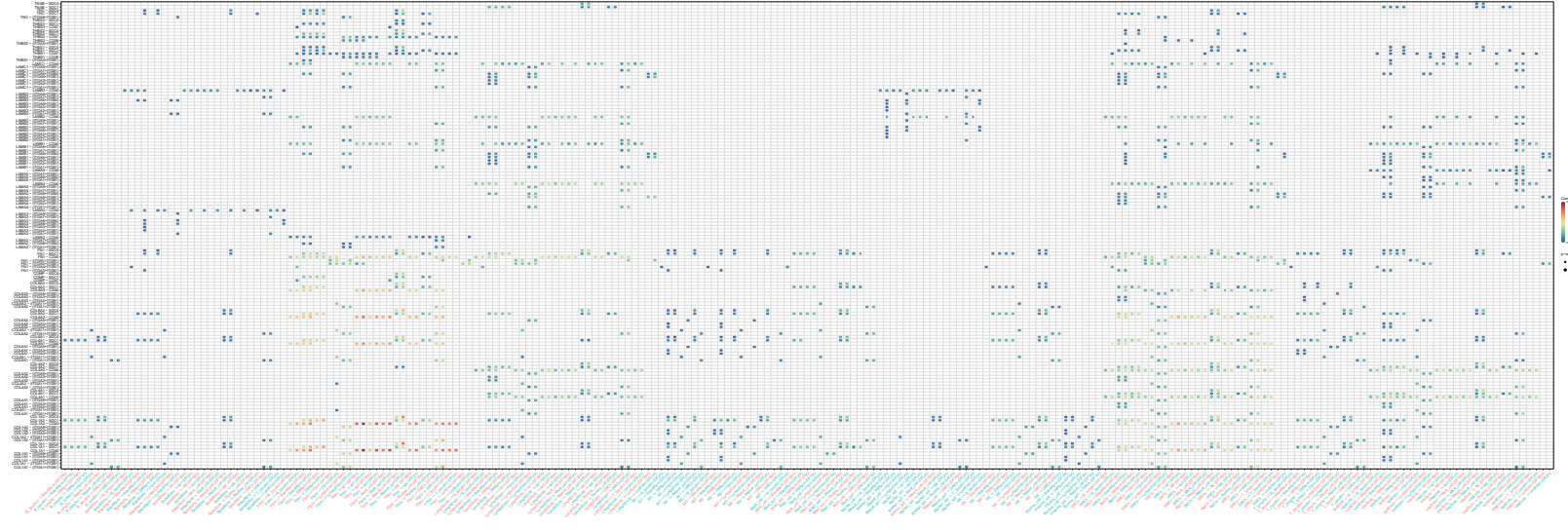

Up

Supplement: S9 Fig — (A) Cell origins of downregulated and upregulated ligand-receptor pairs during the Unhealing process. Blue represents cells from the DFUNH group, and red represents cells from the DS group. (B) Cell origins of downregulated and upregulated ligand-receptor pairs during the Healing process. Blue represents cells from the DFUH group, and red represents cells from the DFUNH group. (PDF) [file pone.0306248.s009.pdf]

A

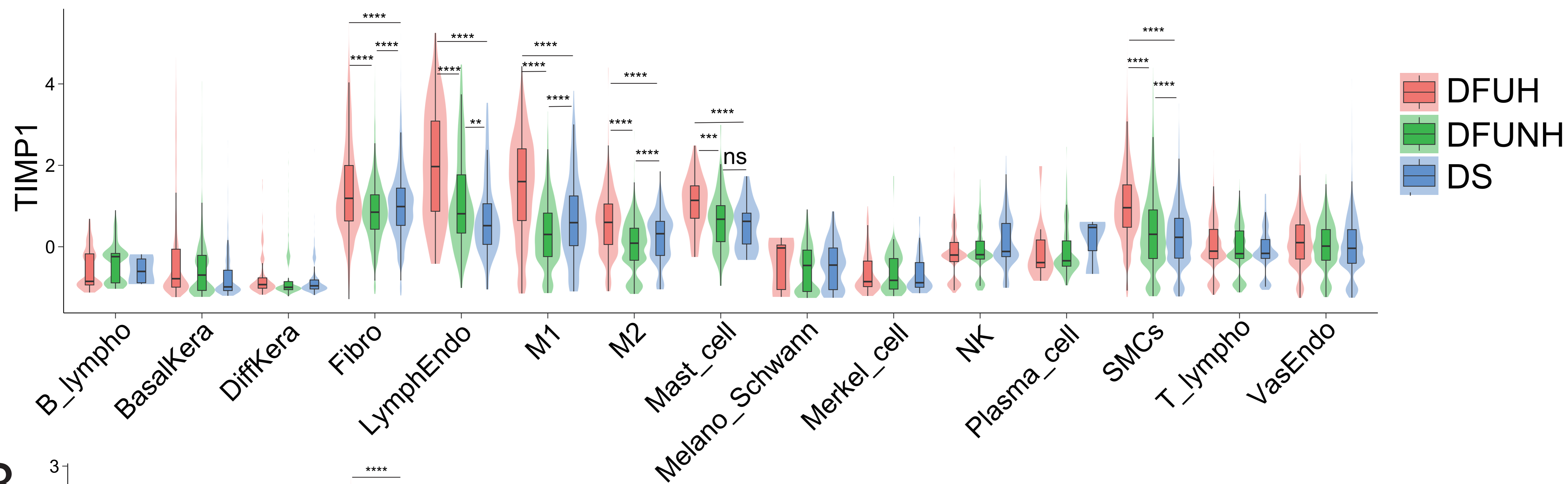

B

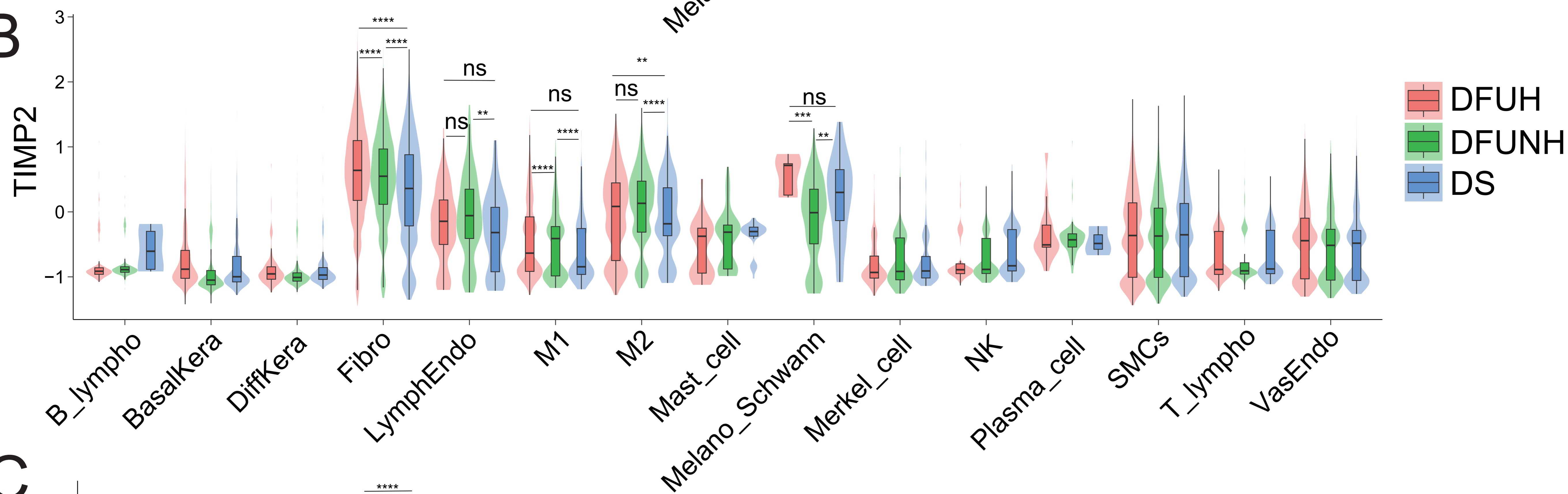

C

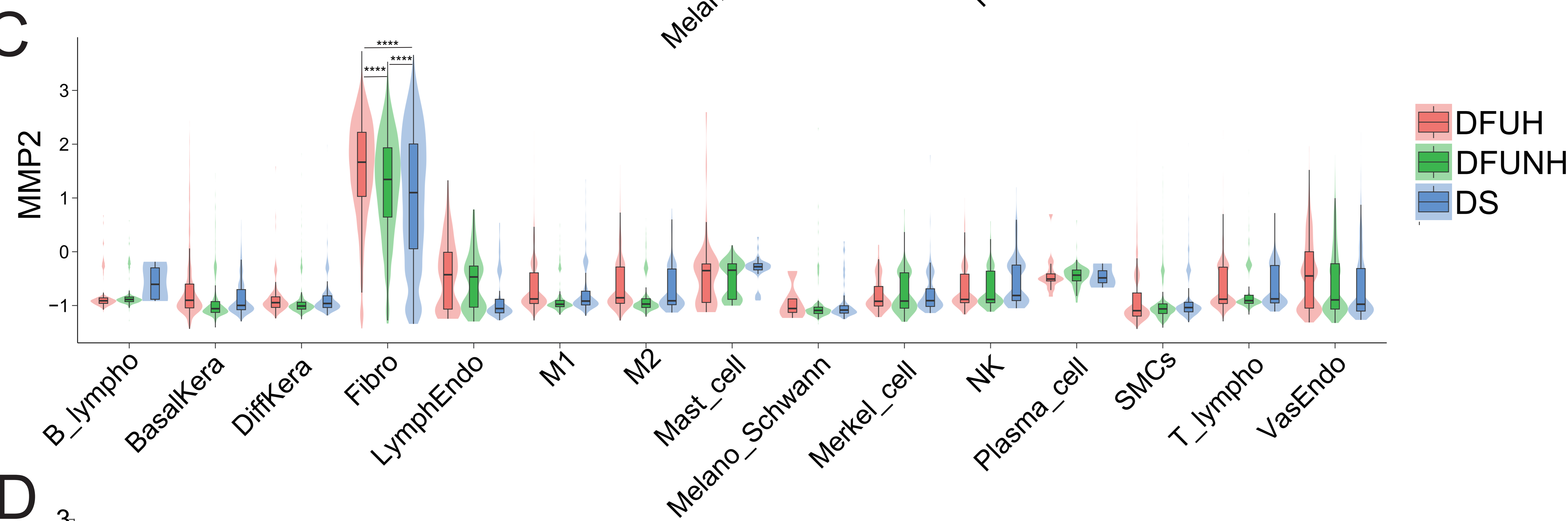

D

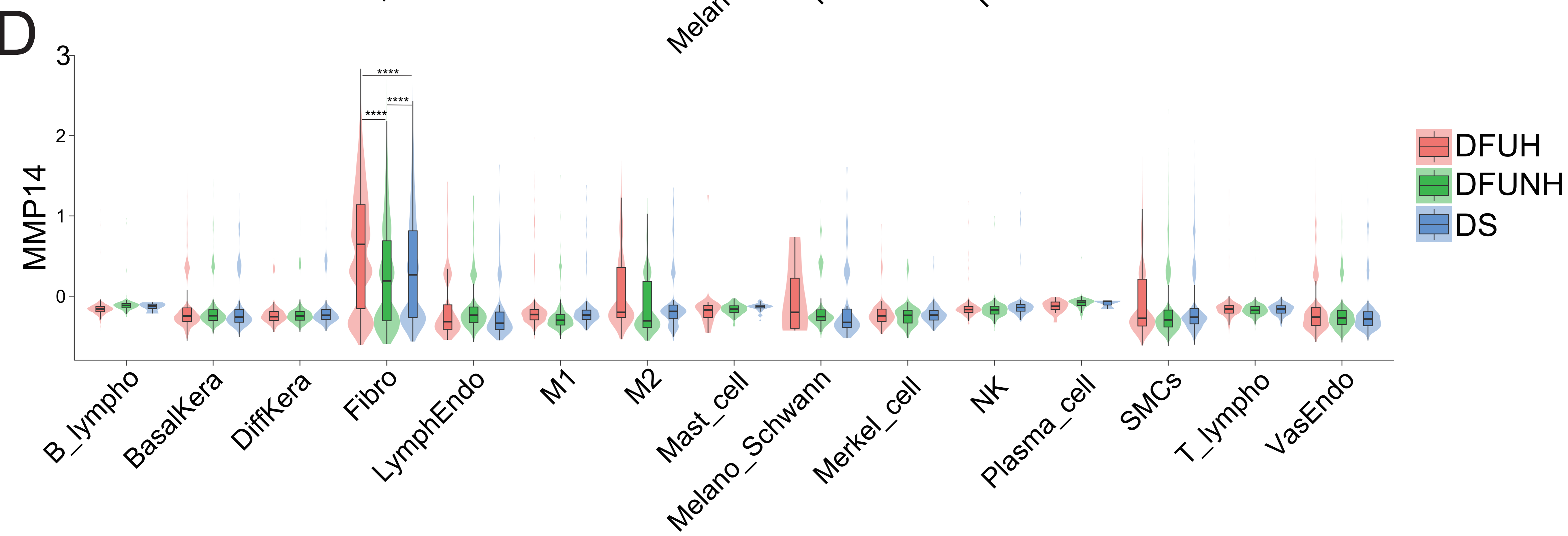

Supplement: S10 Fig — Expression patterns of TIMP1 (A), TIMP2 (B), MMP2 (C) and MMP14 (D) in various cell types within the skin tissue of the three clinical groups. *, P < 0.05; **, P < 0.01; P < 0.001; ****, P < 0.0001. (PDF) [file pone.0306248.s010.pdf]
