# Supplementary material for: Combined analysis of single-cell sequencing and bulk transcriptome sequencing reveals new mechanisms for non-healing diabetic foot ulcers
Source: PLoS One. 2024 Jul 1;19(7):e0306248. doi: 10.1371/journal.pone.0306248 (PMC11216623; doi:10.1371/journal.pone.0306248)
Supplement: S6 Fig — Cell distribution of unhealing (A, D), healing (B, E), and rescue DEGs (C, F) in the skin (A, B, C) and peripheral blood (D, E, F). The gray outer circle represents the union of unhealing, healing, and rescue DEGs, while the different solid colored circles inside the circle represent each distinct cell type. Each cell type is connected to its corresponding DEGs by internal lines in the network. The bar chart on the right side of each cell-network diagram shows the number of DEGs contained in each cell type. (PDF) [file pone.0306248.s006.pdf]

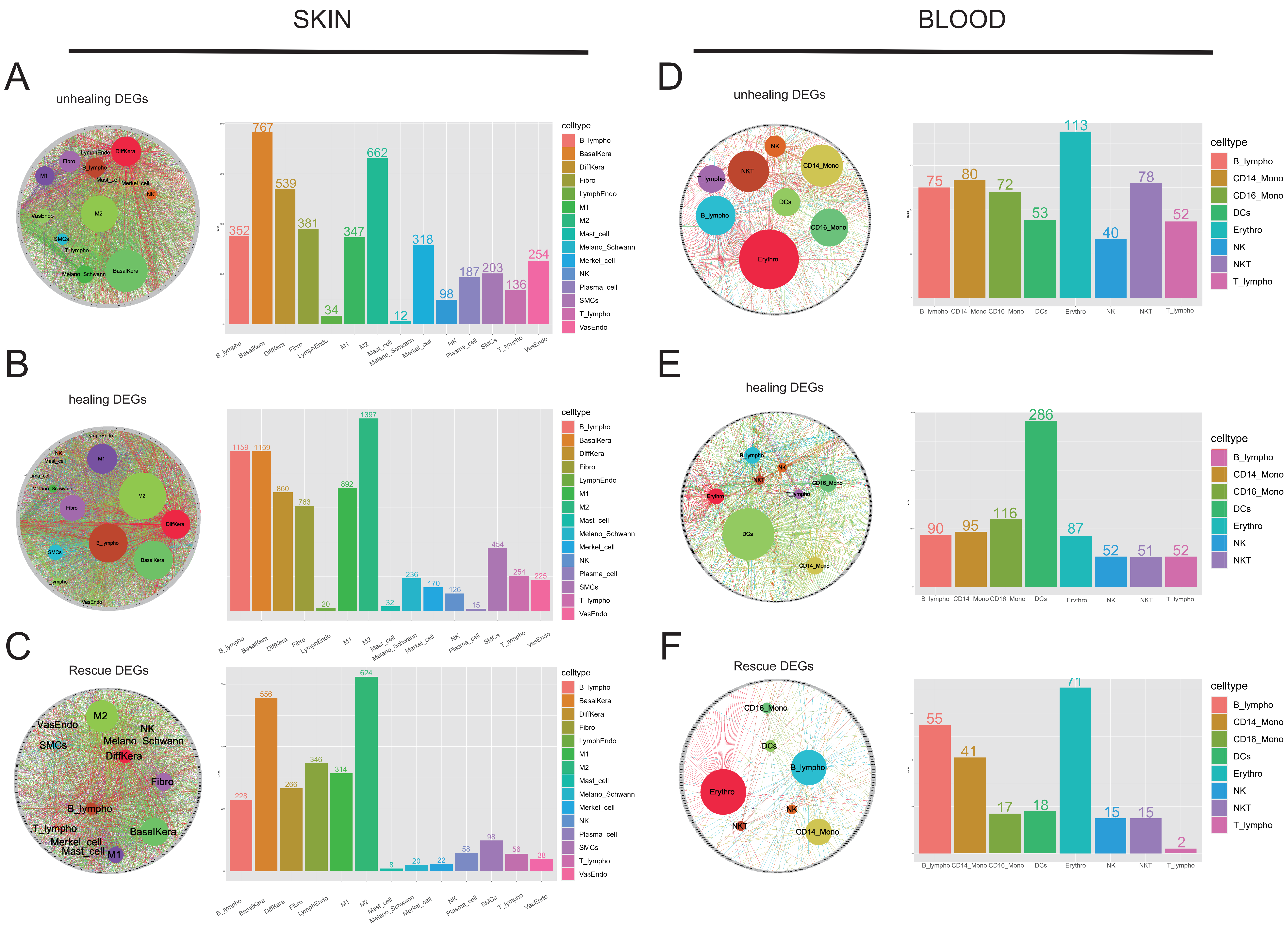
SKIN
BLOOD
A
unhealing DEGs
B
healing DEGs
C
Rescue DEGs
D
unhealing DEGs
E
healing DEGs
F
Rescue DEGs
celltype
B_lympho
BasalKera
DiffKera
Fibro
LymphEndo
M1
M2
Mast_cell
Melano_Schwann
Merkel_cell
NK
Plasma_cell
SMCs
T_lympho
VasEndo
CD14_Mono
CD16_Mono
DCs
Erythro
NKT
352
767
539
381
34
347
662
12
318
98
187
203
136
254
1159
1159
860
763
20
892
1397
32
236
170
126
15
454
254
225
228
556
266
346
314
624
8
20
22
58
98
56
38
75
80
72
53
113
40
78
52
90
95
116
286
87
52
51
52
55
41
17
18
15
15
2
